# SARS-CoV-2 3CLpro as an efficient endopeptidase for fusion tag removal in recombinant protein production

**DOI:** 10.1371/journal.pone.0362685

**Published:** 2026-11-11

**Authors:** Trung-Duc Nguyen, Gia-Bao Do, Phan-Anh Le, Mai-Anh T. Nghiem, Linh T. Ho, Thuy T. Le, Hong-Nhung T. Le, Tuan-Nghia Phan, Hong-Loan T. Nguyen

**Affiliations:** 1 Faculty of Biology, VNU University of Science, Vietnam National University, Hanoi, Vietnam; 2 Key Laboratory of Enzyme and Protein Technology, VNU University of Science, Vietnam National University, Hanoi, Vietnam; São Paulo State University Institute of Biosciences Languages and Exact Sciences: Universidade Estadual Paulista Julio de Mesquita Filho Instituto de Biociencias Letras e Ciencias Exatas, BRAZIL

## Abstract

Fusion tags are widely employed to enhance recombinant protein expression, solubility, and purification. However, efficient tag removal is often necessary to obtain proteins in their native form. In this study, SARS-CoV-2 3C-like protease (3CLpro) was evaluated as a sequence-specific protease for fusion-tag removal using a recombinant fusion protein substrate under standard protein purification conditions. A substrate containing the canonical recognition sequence AVLQ↓SG was efficiently cleaved by 3CLpro, whereas no detectable cleavage was observed for a variant substrate harboring the sequence TVRLQ↓SG. Biochemical characterization demonstrated that 3CLpro maintained cleavage activity across a range of pH values and temperatures and in the presence of reagents commonly used for protein purification and stabilization. 3CLpro-mediated cleavage was successfully integrated into different fusion-protein processing workflows, including in-solution cleavage, on-column cleavage, and dialysis-coupled cleavage. To further evaluate the practical utility of 3CLpro, a modular expression vector (pET_RPLa) incorporating a 3CLpro cleavage site was constructed and validated using the light chain of botulinum neurotoxin serotype A (LC-BoNT/A) as a model protein. Following expression and purification, the fusion protein (6 × His-GST-AVLQSG-LC-BoNT/A) retained SNAP25-cleaving activity and was efficiently processed by 3CLpro. Together, these results support the potential use of SARS-CoV-2 3CLpro as a versatile sequence-specific protease for fusion-tag removal and recombinant protein processing.

## Introduction

Recombinant protein expression, particularly in bacterial hosts, is a well-established technology. However, obtaining soluble, properly folded, and stable proteins, and purifying them efficiently, remain challenging [1,2]. Fusion-tag strategies are therefore widely used to enhance protein solubility and stability and simplify purification workflows. Common fusion partners, including glutathione S-transferase (GST), maltose-binding protein (MBP), thioredoxin (Trx), N-utilization substance A (NusA), and small ubiquitin-like modifier (SUMO), have been shown to increase protein yield and promote soluble expression [1]. When combined with appropriate signal peptides, fusion tags can also support periplasmic targeting in *E. coli* [1,3,4]. Fusion tags may, nevertheless, interfere with the native structure, biological activity, or downstream applications of the target protein [2,5,6], making their removal necessary in many cases.

Site-specific proteases are widely used because of their high selectivity and predictable cleavage patterns [6]. The selection of an appropriate protease depends on multiple factors, including sequence specificity, catalytic efficiency, cost, compatibility with purification conditions, residual amino acids after cleavage, and the ease of removing the cleaved tag and protease after digestion. Several classical serine proteases such as Factor Xa, enterokinase, and thrombin have been used for tag removal, but their specificity remains a concern [7,8].

Viral proteases, particularly tobacco etch virus (TEV) protease and human rhinovirus (HRV) 3C protease, are widely used for tag removal because of their high sequence specificity and compatibility with diverse buffer conditions [5]. These enzymes belong to the chymotrypsin-like protease family, which contains a catalytic cysteine residue. TEV protease typically recognizes sequences containing Gln at the P1 but has relatively slow catalytic turnover and often requires prolonged incubation to achieve efficient cleavage [8,9]. HRV 3C protease has very high specificity at the P1 and P1′ positions, recognizing only Gln/Glu at P1 position, and Gly/Ala/Cys/Ser at P1′ position, respectively [10]. More recently, a consensus-engineered viral protease (Con1) has been reported as an affinity-tag removal enzyme with high substrate specificity and broad P1′ residue tolerance [9]. Recognition sequences for a given protease may be present within the target protein itself, leading to unintended cleavage. As a result, no single protease is universally suitable for removing fusion tags from all recombinant proteins. These limitations highlight the value of expanding the repertoire of site-specific proteases with distinct substrate specificities and biochemical properties, thereby providing greater flexibility in recombinant protein production and purification.

The SARS-CoV-2 3C-like protease (3CLpro, also known as Mpro) is a cysteine protease that processes viral polyproteins at conserved recognition sequences, with Gln at the P1 position. Among these sites, the AVLQ↓SG motif has been reported to be efficiently cleaved by 3CLpro. Its substrate recognition and catalytic mechanism have been extensively characterized [11–15]. 3CLpro possesses several properties that are potentially advantageous for fusion-tag removal, including defined substrate specificity, compatibility with diverse buffer conditions, and catalytic efficiency reported to be approximately ten-fold higher than TEV protease [4,16,17]. Previous studies have demonstrated the feasibility of applying 3CLpro to recombinant protein processing [4,18]. An engineered variant of SARS-CoV 3CLpro was shown to recognize a modified cleavage sequence for fusion-tag removal [18]. More recently, SARS-CoV-2 3CLpro has been used to mediate intracellular cleavage of fusion proteins in *E. coli* [4]. However, systematic evaluation of recombinant SARS-CoV-2 3CLpro for *in vitro* fusion-protein cleavage under purification-relevant conditions and across different processing formats remains limited. Furthermore, the integration of validated 3CLpro cleavage sequences into expression platforms for recombinant protein production has not been widely explored.

In this study, we evaluated SARS-CoV-2 3CLpro-mediated cleavage of a recombinant fusion protein under a range of purification-relevant conditions and using different cleavage formats. We further developed and validated a modular *E. coli* expression vector incorporating a 3CLpro recognition sequence for recombinant protein production and site-specific tag removal.

## Materials and methods

### Plasmid construction

To generate a recombinant substrate for evaluating the endopeptidase activity of SARS-CoV-2 3CLpro, an expression construct encoding 6 × His-GST-AVLQSG-EYFP was designed using the pETM33-Nsp5-Mpro (plasmid # 156475). In this design, the coding sequence for EYFP was fused downstream of the 3CLpro recognition sequence AVLQSG, allowing proteolytic release of native EYFP after cleavage between the P1 glutamine and P1′ serine residues. The DNA fragment encoding AVLQSG-EYFP was amplified from pET28CLY1 plasmid (plasmid # 21760) using the following primers: EYFP_Fw: 5’- *CCATGG* AC *GCG GTACTGCAGAGCGGC*ATGGTGAGCAAGGGCGAGGAG (the NcoI cleavage site is underlined, and the 18 nucleotides coding for the AVLQSG are in italic) and the EYFP_Rv: 5’*GAATTC*TTACTTGTACAGCTCGTCCATGCC (the EcoRI cleavage site is underlined). The PCR product was cloned into the cloning vector pJET1.2/blunt (Thermo Scientific, USA) to create pJET-*AVLQSG*-*EYFP* vector. Subsequently, both pJET-*AVLQSG*-*EYFP* and pETM33-Nsp5-Mpro were digested with NcoI and EcoRI, and *AVLQSG-EYFP* fragment was ligated into the linearized pETM33 backbone. The resulting plasmid, designated pETM33-*AVLQSG-EYFP*, encodes a fusion protein consisting of an N-terminal 6 × His-GST tag followed by the engineered cleavage site and EYFP. The final construct encodes the 6 × His-GST-AVLQSG-EYFP fusion protein, with a molecular mass of approximately 56 kDa, and was used as the substrate for proteolytic cleavage assays.

To evaluate the sequence specificity of 3CLpro, a variant plasmid, pETM33-*TVRLQSG-EYFP* was constructed using the same cloning strategy as described for pETM33-*AVLQSG-EYFP*. Briefly, the TVRLQSG sequence was introduced by replacing the forward primer used for amplification of the AVLQSG fragment, while pET28CLY1 was used as the PCR template. The resulting construct was verified by DNA sequencing.

### Expression and purification of 6 × His-GST-AVLQSG-EYFP

pETM33-*AVLQSG-EYFP* plasmid was transformed into *E. coli* strain BL21-CodonPlus (DE3)-RIL (Novagen, USA). For starter culture, a single colony was inoculated into 5 mL of LB medium containing 100 μg/mL kanamycin and incubated overnight at 37°C with shaking at 200 rpm. The overnight culture was diluted into 100 mL of LB medium in 500-mL flasks to have an initial OD_600_ of 0.05 and incubated at 37°C until the OD_600_ reached approximately 0.7. Isopropyl β-D-1-thiogalactopyranoside (IPTG) was then added to a final concentration of 0.5 mM, and the cell culture was incubated at 18°C for 20 hours. Cells were harvested by centrifugation (Sigma 3K, Sigma) at 6000 × g for 10 minutes at 4^o^C. The pellets were resuspended in 1 mL of PBS 1x pH 7.4 or lysis buffer (10 mM Na_2_PO_4_, 1.8 mM KH_2_PO_4_, 140 mM NaCl, 2.7 mM KCl) (buffer A) containing 5 mM imidazole. Cell disruption was performed by sonication on ice (8 cycles of 30 s pulse and 30 s rest). Lysates were clarified by centrifugation at 12,000 × g for 20 minutes at 4°C. The soluble fraction containing total protein was collected for analysis. The expression level of protein was evaluated by SDS-PAGE.

6 × His-GST-AVLQSG-EYFP was purified from the lysate of BL21-CodonPlus (DE3)-RIL cells harboring the pETM33-*AVLQSG-EYFP* plasmid using a manually packed Ni-Sepharose column (GE Healthcare) operated by gravity flow. The lysate was loaded onto the column, pre-equilibrated with buffer A. The unbound fractions were washed with buffer A containing 5 mM and 50 mM imidazole until the absorbance at 280 nm (A_280_) reached baseline. Proteins bound to the Ni-Sepharose column were eluted in a single step with buffer A containing 250 mM imidazole. The recombinant EYFP-positive fractions were pooled and dialyzed against buffer A (10% glycerol) for 24 h prior to downstream enzymatic assays. The collected protein fractions were analyzed by SDS-PAGE on a 12% gel. Protein concentration was determined by the Bradford assay with BSA as a standard [19]. 6 × His-GST-TVRLQSG-EYFP was expressed and purified using the same procedure as described for 6 × His-GST-AVLQSG-EYFP.

Recombinant SARS-CoV-2 3CLpro variants, au3CLpro (designated as 3CLpro in this study) and com3CLpro were prepared as described previously [17].

### Cleavage assay and analysis of recombinant 3CLpro activity

The cleavage activity of 3CLpro was evaluated using the recombinant fusion substrate 6 × His-GST-AVLQSG-EYFP, which contains the 3CLpro recognition sequence AVLQ↓SG. Specific cleavage at this site by 3CLpro generates an N-terminal 6 × His-GST-containing fragment (~29 kDa) and a C-terminal EYFP-containing fragment (~27 kDa). Cleavage reactions were performed by incubating 3 μg of substrate with 0.15–1.0 μg of purified 3CLpro (corresponding to substrate-to-enzyme ratios of 20:1–3:1, m/m) in a reaction buffer containing 50 mM Tris-HCl (pH 7.0), 150 mM NaCl and 1 mM DTT (buffer B) in a total volume of 25 μL. Reactions were incubated at 4°C, 25°C and 37°C. At defined time points (3, 6, and 18 h), reactions were stopped and mixed with 5 × SDS-PAGE loading buffer (312.5 mM Tris-HCl, pH 6.8 containing 10% (m/v) sodium dodecyl sulfate, 25% (v/v) β-mercaptoethanol, 50% (v/v) glycerol and 0.05% (m/v) bromophenol blue) and stored at −20°C until analyzed.

Cleavage products were analyzed by SDS-PAGE and visualized by Coomassie Brilliant Blue R-250 staining. Protein cleavage was primarily assessed by visual inspection of SDS-PAGE band patterns, supported by semiquantitative densitometric analysis using ImageJ software (NIH, USA). Band intensities were measured for the full-length substrate band (~56 kDa) and the two expected cleavage-product bands (~29 and ~27 kDa). The percentage of cleavage was estimated by dividing the combined pixel intensity of the cleavage-product bands by the total pixel intensity of the full-length substrate and cleavage-product bands [20]. For the evaluation of reagent effects, relative cleavage (%) was calculated as the ratio of the estimated cleavage percentage in the presence of each reagent to that of the corresponding control without the reagent. The resulting values were used for relative comparison of cleavage efficiency among the tested conditions.

The effect of reagents commonly used in protein purification and stabilization on 3CLpro activity was evaluated at 4^o^C. The reagents tested included: (1) compounds used for the elution of polyhistidine-tagged (imidazole), GST-tagged (reduced glutathione), MBP-tagged (maltose) proteins from the relevant affinity resins; (2) salts (NaCl, KCl, CaCl_2_, MgCl_2_, MgSO_4_ and ZnSO_4_; (3) protein stabilizers, detergents, chelators and inhibitors (glycerol, Tween-20, Triton X-100, EDTA, PMSF and solvent-matched control included); (4) denaturants (urea, guanidine-HCl, SDS); and (5) reducing agents (DTT, β-mercaptoethanol [β-ME]). The reagents used in these assays were obtained from the following suppliers: imidazole from Serva (Germany); NaCl, KCl, CaCl_2_, MgCl_2_, urea, and SDS from Merck (Germany); reduced glutathione, glycerol, Triton X-100, PMSF, DTT, β-mercaptoethanol, and ZnSO_4_ from Sigma-Aldrich (USA); and MgSO_4_ and EDTA from Prolabo (France). Cleavage reactions were performed using the fusion substrate 6 × His-GST-AVLQSG-EYFP and 3CLpro at a substrate-to-enzyme ratio of 10:1 (m/m) in buffer B. After incubation, the reaction was terminated by the addition of SDS-PAGE sample buffer and samples were stored at −20°C until use. The 3CLpro cleavage efficiency was estimated visually from SDS-PAGE gels stained with Coomassie Brilliant Blue [5].

### Fusion tag removal using 3CLpro

Fusion tag removal using 3CLpro was performed with the recombinant substrate 6 × His-GST-AVLQSG-EYFP under three purification formats: in-solution cleavage, on-column cleavage, and dialysis-coupled cleavage.

For in-solution cleavage, 0.3 mg of purified 6 × His-GST-AVLQSG-EYFP was incubated with 0.1 mg of 3CLpro in buffer B at 37°C for 3 h. The reaction mixture was then applied onto a Ni-Sepharose column (1x1 cm) pre-equilibrated with buffer B. The flow-through fraction containing cleaved EYFP was collected, whereas His-tagged proteins, including 6 × His-GST and 3CLpro, remained bound to the resin and were subsequently eluted with buffer B containing 250 mM imidazole.

For on-column cleavage, the soluble lysate of *E. coli* BL21-CodonPlus(DE3)-RIL cells expressing 6 × His-GST-AVLQSG-EYFP was first loaded onto a Glutathione-Sepharose column (1x1 cm) equilibrated with buffer A. After washing to remove unbound proteins, the column was further equilibrated with buffer B. 0.1 mg of purified 3CLpro was then loaded directly onto the column and kept at 37°C for 3 h to allow cleavage. The flow-through fraction was collected and subsequently applied to a Ni-Sepharose column (to retain His-tagged proteins) and tag-free EYFP fragment was collected in the flow-through fraction.

For dialysis-coupled cleavage, 0.3 mg of purified 6 × His-GST-AVLQSG-EYFP, eluted from Ni-Sepharose in buffer A containing 250 mM imidazole, was mixed with 0.1 mg of 3CLpro and transferred into dialysis tubing. The sample was dialyzed against buffer B at 4°C for 18 h, with 3 changes of buffer B. After dialysis, the reaction mixture was applied onto a Ni-Sepharose column as described above to separate tag-free EYFP fragment from His-tagged proteins.

Protein samples from each purification step of in-solution cleavage, on-column cleavage, and dialysis-coupled cleavage were analyzed by SDS-PAGE.

### Construction of 3CLpro-based expression vector

The 3CLpro-based expression vector (pET_RPLa) was constructed using the pETM33-Nsp5-Mpro backbone (Addgene #156475) by introducing the canonical 3CLpro cleavage sequence (AVLQ↓SG) between the GST coding region and the multiple cloning site (MCS). This design allows target genes to be inserted downstream of the 3CLpro cleavage site, enabling site-specific removal of the N-terminal fusion tag by recombinant 3CLpro.

The GST-AVLQSG fragment was amplified by PCR using Q5® High-Fidelity DNA polymerase (NEB, USA) with primers designed to incorporate both the 3CLpro recognition sequence and overlapping regions for Gibson Assembly. Specifically, Inserta_Fw **(**5′-*ACAACACTAGTAGCAATTCC*ATGTCCCCTATAC-3′) comprises a 5′ overlap region complementary to the vector backbone, followed by a sequence corresponding to the 5′ end of the GST coding region. The reverse primer Inserta_Rv (5′-TCGACGGAGCTCGAATTCCTGGATCCGCCGCTCTGCAGTACCGCACGTTTTGGAGGATGG-3′) comprises, from 5′ to 3′, an overlap region for Gibson Assembly, restriction sites (SacI, EcoRI, and BamHI) corresponding to the MCS, the coding sequence for the 3CLpro recognition motif (AVLQSG), and a sequence complementary to the 3′ end of the GST coding region. In parallel, the pETM33-Nsp5-Mpro vector was linearized by PCR using the primers Opena_Fw (5′-AGGAATTCGAGCTCCGTCGA-3′) and Opena_Rv (5′-GGAATTGCTACTAGTGTTGTGATGGTG-3′), which introduce terminal overlaps complementary to the insert fragment. PCR products were treated with DpnI (NEB, USA) to remove template DNA, purified, and assembled using Gibson Assembly (NEB, USA). The resulting construct contains a modular expression cassette organized as 6 × His-GST-AVLQSG-MCS-6 × His, in which the MCS region enables insertion of target genes while preserving the correct reading frame for 3CLpro-mediated cleavage. The plasmid was transformed into *E. coli* TOP10 cells, and recombinant clones were identified by colony PCR using T7 promoter and terminator primers and confirmed by Sanger sequencing.

### Cloning, expression, and purification of the light chain of botulinum neurotoxin serotype A using pET_RPLa

The gene encoding the light chain of botulinum neurotoxin serotype A (LC-BoNT/A) was amplified by PCR from plasmid pBN3-BoNT/A-LC (Addgene #31602) using the following primers:

LC-BoNT/A_Fw: 5′ GAATTCCATGCCATTTGTTAATAAACAATTTAATTATAAAGATCC-3′ (the EcoRI cleavage site is underlined) and LC-BoNT/A_Rv: 5′-CTCGAGAGATCCACGCGGAACCAG-3′ (the XhoI cleavage site is underlined). The PCR product (~1.3 kb) was cloned into pJET1.2/blunt vector (Thermo Scientific, USA). The *LC-BoNT/A* insert was subsequently subcloned into the pET_RPLa vector using EcoRI and XhoI restriction sites to generate pET_RPLa-*LC-BoNT/A*. For protein expression, the recombinant plasmid was transformed into *E. coli* BL21(DE3). Cells were grown at 37°C in LB medium supplemented with kanamycin (100 µg/mL) and chloramphenicol (34 µg/mL) to reach an OD_600_ of 0.6–0.8 and induced with 0.25 mM IPTG. Expression was carried out at 16°C for 20 h. Cells were harvested, resuspended in PBS (pH 7.4) containing 10 mM imidazole, and lysed by sonication on ice for eight 30-s cycles with 30-s intervals between cycles. The lysates were centrifuged at 13,000 × g for 20 min at 4°C, and the resulting clear supernatants were pooled and applied to a Ni-Sepharose affinity column pre-equilibrated with the same buffer. The column was washed with buffer containing increasing concentrations of imidazole (10–50 mM), and the fusion protein was eluted with 250 mM imidazole. Eluted fractions were analyzed by SDS-PAGE, and the purified protein was subjected to dialysis against PBS buffer supplemented with 10% glycerol at 4°C. Protein samples were stored at −80°C until use.

### Proteolytic activity assay of recombinant LC-BoNT/A using SNAP25 substrate

The proteolytic activity of recombinant LC-BoNT/A was evaluated using the recombinant substrate ECFP-SNAP25(141−206)-mVenus-6 × His (SNAP25 substrate)**,** as previously described with minor modifications [21]. The reaction mixture (20 μL) contained reaction buffer (20 mM Na-HEPES, pH 7.4, 1 mM DTT), 0.3 μg of recombinant LC-BoNT/A, 7 μg of recombinant SNAP25 substrate. The reaction was incubated at 37°C for 3 h and terminated by addition of SDS sample buffer followed by heating at 95°C for 5 min. Reaction products were analyzed by 12% SDS-PAGE**.** Cleavage of the approximately 64-kDa SNAP25 substrate generated two fragments of approximately 34 kDa and 30 kDa**,** corresponding to the expected BoNT/A cleavage products. The negative control reactions were performed using heat-inactivated enzyme (incubated at 95°C for 5 min) or in the presence of EDTA.

## Results

### Expression of recombinant 6 × His-GST-AVLQSG-EYFP protein as a substrate for 3CLpro activity assay

To generate a substrate for evaluating 3CLpro endopeptidase activity, the fusion protein 6 × His-GST-AVLQSG-EYFP was expressed in *E. coli* strain BL21-CodonPlus(DE3)-RIL using the pETM33 expression system. As shown in Fig 1, induction with IPTG resulted in the appearance of a prominent protein band at approximately 56 kDa on SDS-PAGE (Fig 1A, lane 2), corresponding to the expected size of the fusion protein. This band was specifically recognized by an anti-His-tag antibody (Fig 1B, lane 2), whereas in the absence of IPTG, the protein was expressed at a lower level (Fig 1A and 1B, lane 1). Noticeably, the recombinant fusion protein was detected more prominently in the soluble fraction (Fig 1A and 1B, lane 3), with only a minor proportion detected in the insoluble fraction (Fig 1A and 1B, lane 4), indicating that the recombinant fusion protein was expressed mainly in a soluble form.

**Fig 1:**
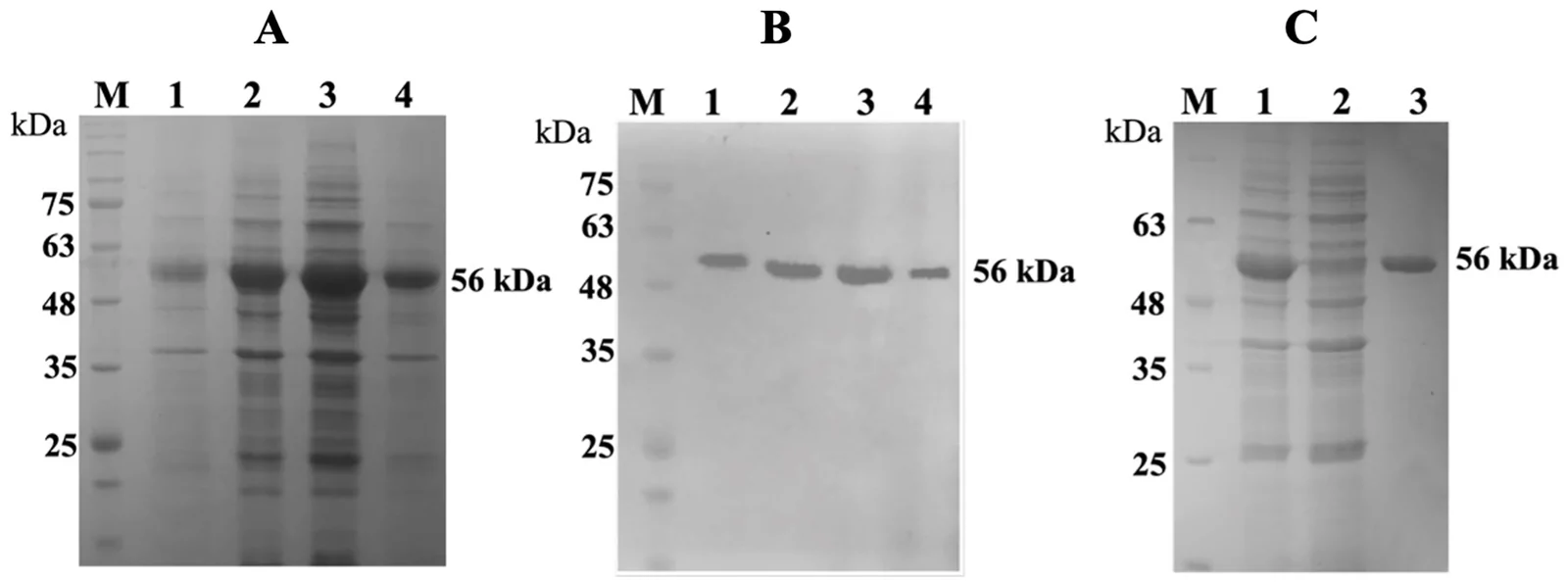
Expression and purification of 6 × His-GST-AVLQSG-EYFP protein. **(A)** SDS-PAGE and **(B)** Western blotting analyses of 6 × His-GST-AVLQSG-EYFP expression in BL21-CodonPlus(DE3)-RIL harboring pETM33-*AVLQSG-EYFP*. Molecular mass marker (lane M); the whole-cell lysates from non-induced and IPTG-induced cultures (lanes 1 and 2, respectively); soluble and insoluble fractions of the cell extract were obtained after sonication (lanes 3 and 4, respectively). SDS-PAGE analysis of purified 6 × His-GST-AVLQSG-EYFP **(C)**. Molecular mass marker (lane M), lysate (lane 1), fraction unbound to Ni-Sepharose column (lane 2), fraction eluted from the Ni-Sepharose column (lane 3).

6 × His-GST-AVLQSG-EYFP from the lysate of BL21-CodonPlus(DE3)-RIL cells harboring pETM33-*AVLQSG-EYFP* was purified to near homogeneity by Ni-Sepharose affinity chromatography, as shown by the predominant band on SDS-PAGE (Fig 1C, lane 3).

### Specific cleavage of 6 × His-GST-AVLQSG-EYFP by 3CLpro

The cleavage activity of 3CLpro was evaluated using the recombinant substrate 6 × His-GST-AVLQSG-EYFP containing the canonical recognition sequence AVLQ↓SG. Cleavage products were analyzed by SDS-PAGE and Western blotting. As shown in Fig 2A, 3CLpro (~34 kDa) alone (lane 1) and the full-length substrate (~56 kDa) alone (lane 2) showed no cleavage products. In contrast, incubation of the substrate with 3CLpro generated two distinct protein bands of approximately 29 and 27 kDa (lane 3), corresponding to the expected cleavage products N-terminal GST-containing fragment and C-terminal EYFP-containing fragment, respectively. No detectable cleavage was observed when substrate was incubated with inactivated 3CLpro (lane 4) or in the presence of GC376, a specific 3CLpro inhibitor (lane 5). To further examine cleavage specificity, com3CLpro (~62 kDa), a recombinant protein lacking the AVLQSG recognition sequence [17], was analyzed alone (lane 6) and after incubation with 3CLpro (lane 7). No detectable cleavage of com3CLpro was observed under these conditions.

**Fig 2:**
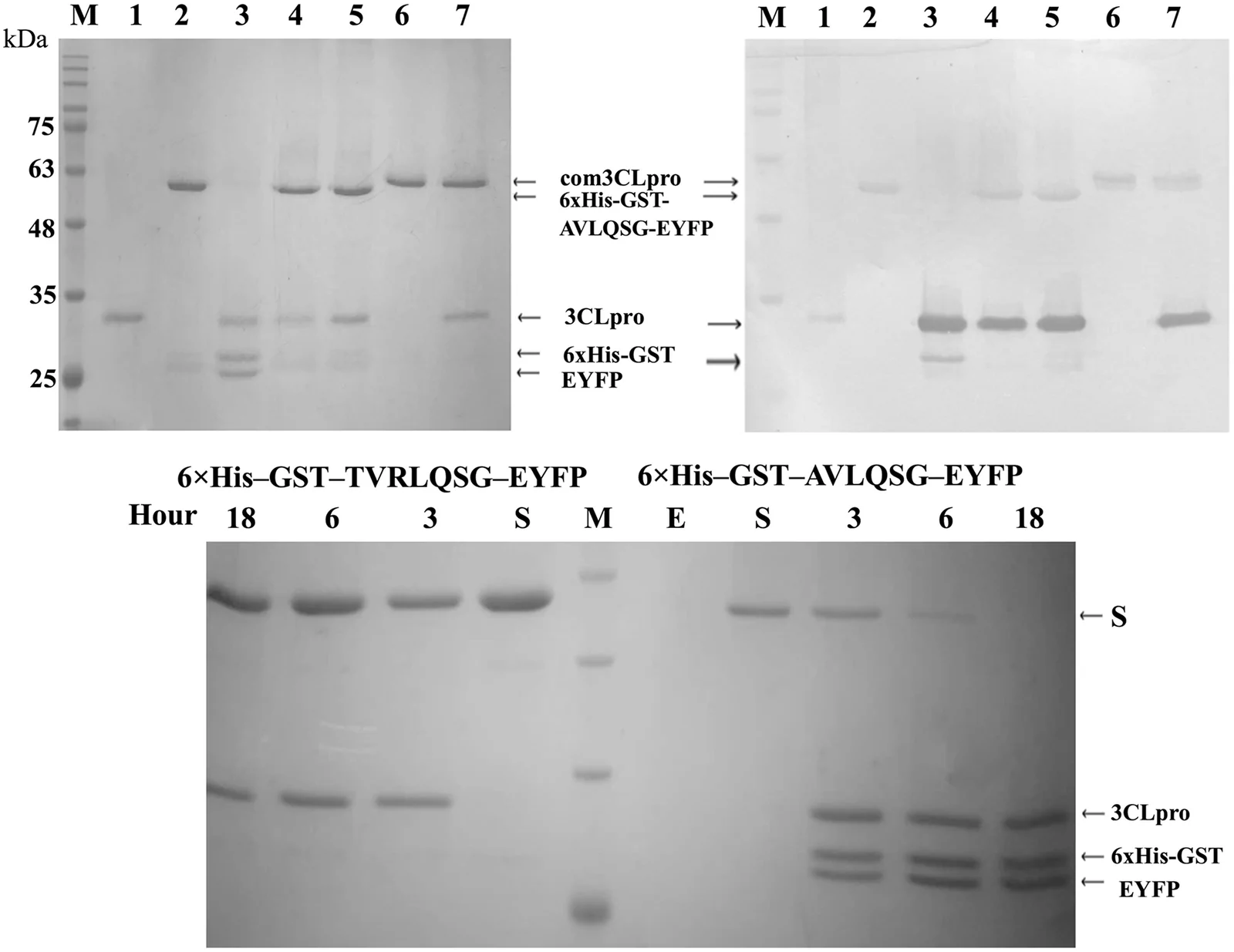
Specific cleavage of fusion substrates by 3CLpro and sequence-dependent substrate recognition. **(A)** SDS-PAGE and **(B)** Western blotting analysis of cleavage products. Molecular mass marker (lane M); purified 3CLpro (lane 1); substrate (6 × His-GST-AVLQSG-EYFP) (lane 2); cleavage reaction of substrate with 3CLpro (lane 3); cleavage reaction with heat-inactivated 3CLpro (95°C, 10 min) (lane 4); cleavage reaction in the presence of the 3CLpro inhibitor GC376 (10 µM) (lane 5); com3CLpro (lane 6); reaction of 3CLpro with com3CLpro (lane 7). **(C)** SDS-PAGE analysis of cleavage reactions using the variant substrate 6 × His-GST-TVRLQSG-EYFP. Cleavage reactions were performed under the same conditions as in (A) and analyzed at different incubation times. Molecular mass marker (lane M); substrate (lane S without 3CLpro); 3CLpro (lane E); cleavage reactions sampled after 3, 6, and 18 h, respectively (lanes 3, 6, and 18 h).

**Fig 3:**
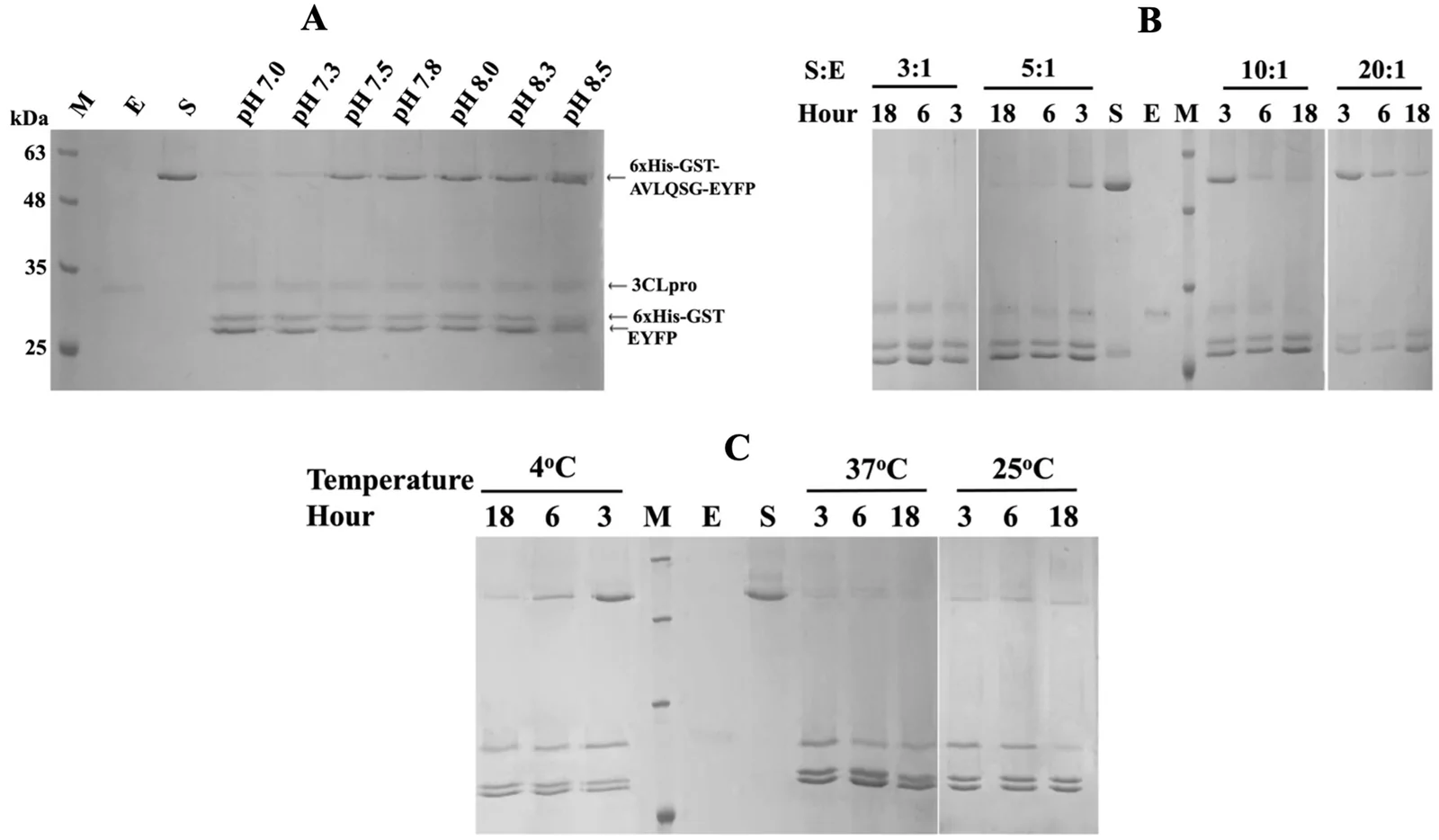
Effects of reaction conditions on 3CLpro-mediated cleavage of the recombinant substrate. **(A)** Effect of pH on 3CLpro cleavage activity. Cleavage reactions were performed under different pH conditions and analyzed by SDS-PAGE. **(B)** Effect of substrate-to-enzyme (S:E) ratio on cleavage efficiency. Reactions were carried out at different S:E ratios (3:1, 5:1, 10:1, and 20:1) and sampled at indicated time points (3, 6, and 18 h). **(C)** Temperature dependence of 3CLpro activity. Cleavage reactions were performed at 4, 25, and 37°C for the indicated incubation times. Molecular mass marker (lane M); substrate (6 × His-GST-AVLQSG-EYFP) (lane S); 3CLpro (lane E).

The results of Western blotting analysis (Fig 2B) showed that a distinct band corresponding to 6 × His-GST-AVLQSG-EYFP (~56 kDa), 6 × His-GST fragment (~29 kDa), com3CLpro (62 kDa) and 3CLpro (~34 kDa) were recognized by anti-6 × His-tag antibody, whereas no band corresponding to the EYFP fragment (~27 kDa, without his tag) was detected. These data reconfirmed the identity of the cleavage products detected by SDS-PAGE.

To further examine the specific cleavage of 3CLPro, a variant fusion substrate 6 × His-GST- TVRLQSG-EYFP (containing the sequence TVRLQSG) was used under the same reaction conditions. In contrast to the 6 × His-GST-AVLQSG-EYFP substrate, the 6 × His-GST-TVRLQSG-EYFP substrate remained intact with no detectable cleavage products observed after 3, 6 and 18 hours incubation of the substrate with 3CLpro (Fig 2C), indicating that 3CLpro-mediated cleavage is highly specific.

Based on its efficient cleavage, high expression level, and soluble expression profile, the 6 × His-GST-AVLQSG-EYFP construct was selected as the model substrate for subsequent experiments to characterize the biochemical properties and reaction conditions of 3CLpro.

### Evaluation of the cleavage activity of 3CLpro under different reaction conditions

The cleavage activity of 3CLpro toward 6 × His-GST-AVLQSG-EYFP was evaluated under different reaction conditions. Among the pH values tested, 3CLpro showed the highest cleavage activity at pH 7.0 (Fig 3A), with activity generally decreasing as the pH increased. Cleavage efficiency increased with enzyme concentration, while a substrate-to-enzyme (S:E) ratio of 10:1 (m/m) still provided substantial cleavage after 18 h (Fig 3B). In Tris-HCl buffer, cleavage was greater at 37°C than at 25°C, but remained substantial even at 4°C (Fig 3C).

The effect of the reagents, which were commonly used in protein extraction, purification and stabilization, on 3CLpro activity was also evaluated. The result (Fig 4) showed that imidazole (10 mM), reduced glutathione (0.1–1 mM), maltose (0.1–10 mM), NaCl (150–800 mM) and KCl (150–600 mM), glycerol (5–50%), Tween-20 (0.1–1%), Triton X-100 (0.05–0.5%), EDTA (1–5 mM), β-mercaptoethanol (1–5 mM) and DTT (1–5 mM) had little or no effect on the cleavage activity of 3CLPro. In contrast, CaCl_2_ (1–5 mM), MgCl_2_ (1–5 mM), MgSO_4_ (1–10 mM), and PMSF (1–5 mM) slightly enhanced cleavage. Conversely, ZnSO_4_ (0.1–10 mM), urea (1–2 M), guanidine hydrochloride (0.5–2 M), and SDS (0.01–0.1%) inhibited 3CLpro-mediated cleavage. At higher concentrations, imidazole (50–200 mM) and reduced glutathione (5–10 mM) also reduced cleavage. In addition, guanidine hydrochloride appeared to induce partial protein precipitation, as evidenced by the reduced intensity of all protein bands observed on SDS-PAGE gels with increasing guanidine hydrochloride concentrations.

**Fig 4:**
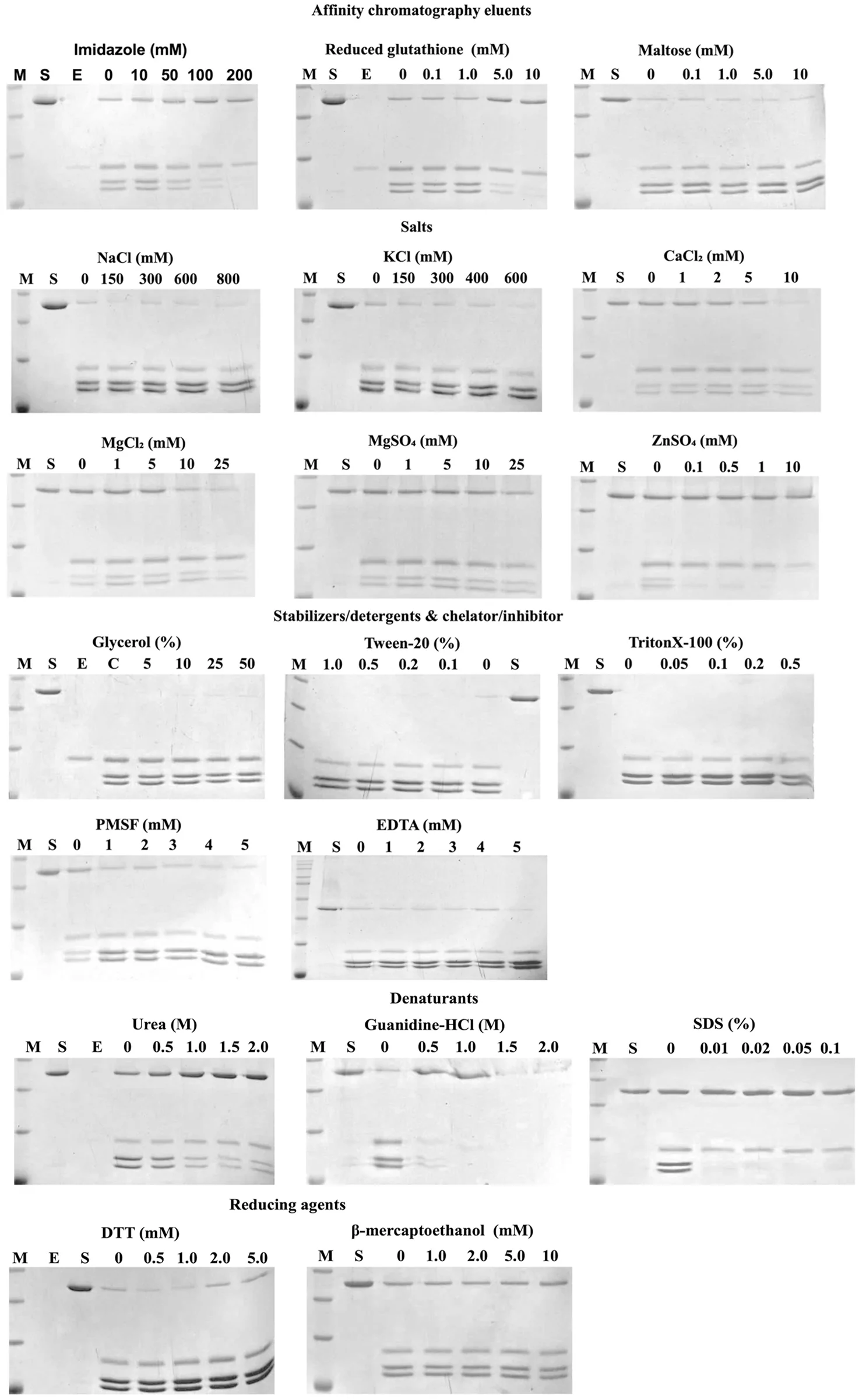
Effects of commonly used protein purification and stabilization reagents on 3CLpro cleavage activity. The effects of affinity chromatography eluents (imidazole, reduced glutathione, and maltose), salts (NaCl, KCl, CaCl_2_, MgCl_2_, MgSO_4_, and ZnSO_4_), stabilizers, detergents, chelator and inhibitor (glycerol, Tween-20, Triton X-100, PMSF, and EDTA), denaturants (urea, guanidine hydrochloride, and SDS), and reducing agents (DTT and β-mercaptoethanol) on 3CLpro-mediated cleavage of the recombinant substrate 6 × His-GST-AVLQSG-EYFP were evaluated at the indicated concentrations. Cleavage products were analyzed by SDS-PAGE. Molecular mass marker (lane M); substrate (6 × His-GST-AVLQSG-EYFP) (lane S); 3CLpro (lane E).

Collectively, these results indicate that 3CLpro retained cleavage activity under a broad range of conditions commonly encountered during recombinant protein purification. Based on the obtained results, a buffer containing 50 mM Tris-HCl (pH 7.0), 150 mM NaCl, 1 mM DTT, 1 mM EDTA and 1 mM PMSF was selected as a suitable reaction condition for efficient cleavage of the fusion substrate.

### Application of 3CLpro for removing tag from recombinant protein under different purification conditions

We next evaluated the efficiency of 3CLpro in removing the fusion tag from the recombinant protein (6 × His-GST-AVLQSG-EYFP) under different purification conditions: in-solution, on-column, and during dialysis.

As shown by SDS-PAGE analysis (Fig 5A), 3CLpro efficiently cleaved the 6 × His-GST-AVLQSG-EYFP fusion protein (~56 kDa) in solution, generating two major products of approximately 29 and 27 kDa (lane 5), corresponding to the expected GST-containing and EYFP-containing fragments, respectively. Following cleavage, the reaction mixture was applied to a Ni-Sepharose column. The EYFP-containing fragment, which lacks the N-terminal His-tag, did not bind to the resin and was recovered in the flow-through fraction (lane 6). In contrast, the 6 × His-GST-containing fragment and 3CLpro remained bound and were subsequently eluted with buffer B containing 250 mM imidazole (lane 7). This strategy enabled straightforward recovery of tag-free EYFP.

**Fig 5:**
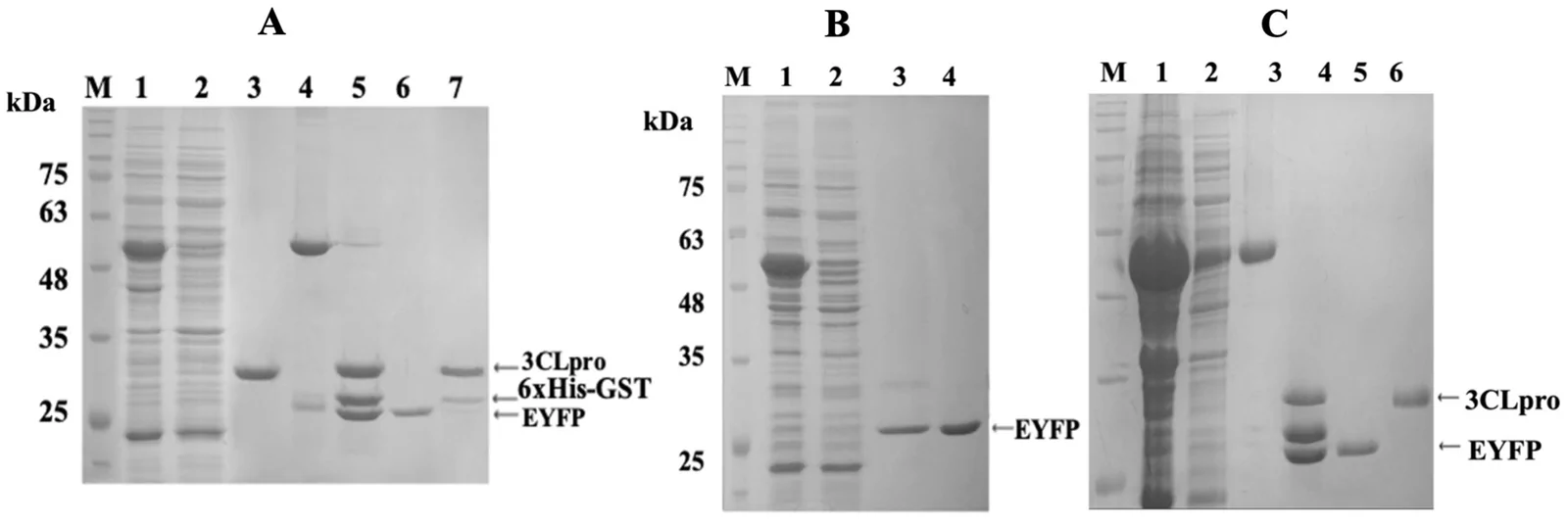
SDS-PAGE of 3CLpro cleaved products of 6 × His-GST-AVLQSG-EYFP protein under in-solution, on-column, and during dialysis cleavage conditions. **(A)** In-solution cleavage. Molecular mass marker (lane M); crude cell lysate (lane 1); flow-through fraction from Ni-Sepharose (lane 2); 3CLpro (lane 3); bound fraction from Ni-Sepharose containing 6 × His-GST-AVLQSG-EYFP (lane 4); cleavage products after incubation with 3CLpro (lane 5); flow-through fraction after Ni-Sepharose purification containing EYFP fragment (lane 6); and bound fraction containing His-tagged proteins (6 × His-GST and 3CLpro) (lane 7). **(B) On-column cleavage.** Molecular mass marker (lane M); crude cell lysate (lane 1); flow-through fraction from Glutathione-Sepharose (lane 2); supernatant collected after on-column cleavage containing the EYFP fragment and 3CLpro (lane 3); and released EYFP-containing fragment after cleavage by 3CLpro (lane 4). **(C)** Cleavage during dialysis. Molecular mass marker (lane M); crude cell lysate (lane 1); flow-through fraction from Ni-Sepharose (lane 2); bound fraction from Ni-Sepharose containing 6 × His-GST-AVLQSG-EYFP (lane 3); cleavage products after incubation with 3CLpro during dialysis (lane 4); flow-through fraction containing EYFP fragment (lane 5); and 3CLpro (lane 6).

In the on-column cleavage approach, 6 × His-GST-AVLQSG-EYFP was first immobilized on Glutathione-Sepharose through its GST tag and subsequently treated with 3CLpro. Cleavage released the EYFP-containing fragment and 3CLpro into the supernatant (Fig 5B, lane 3), while the 6 × His-GST-containing fragment remained bound to the resin. The flow-through fraction was subsequently applied to a Ni-Sepharose column to retain His-tagged 3CLpro, allowing tag-free EYFP to be recovered in the unbound fraction (Fig 5B, lane 4).

Similarly, when 6 × His–GST-AVLQSG-EYFP was incubated with 3CLpro during dialysis, the fusion protein was efficiently cleaved, yielding the same products, 6 × His-GST-AVLQ and SG-EYFP. These cleavage products were subsequently separated and purified by Ni-Sepharose chromatography (Fig 5C, lane 5), demonstrating that 3CLpro-mediated tag removal can be effectively integrated into a dialysis-based purification workflow.

### Development and application of the 3CLpro-based expression vector

By inserting a DNA sequence encoding the 3CLpro cleavage motif (AVLQ↓SG) between the GST-coding region and the multiple cloning site (MCS), we generated a modular open reading frame (ORF) architecture (6 × His-GST-AVLQSG-MCS-6 × His) that enables direct insertion of target genes downstream of the 3CLpro cleavage site (Fig 6A). Consequently, the expressed fusion proteins can be efficiently processed by 3CLpro and readily purified using affinity chromatography. Correct assembly of the vector was confirmed by colony PCR and Sanger sequencing (S2 File), which verified both the successful insertion of the cleavage sequence and preservation of the reading frame.

**Fig 6:**
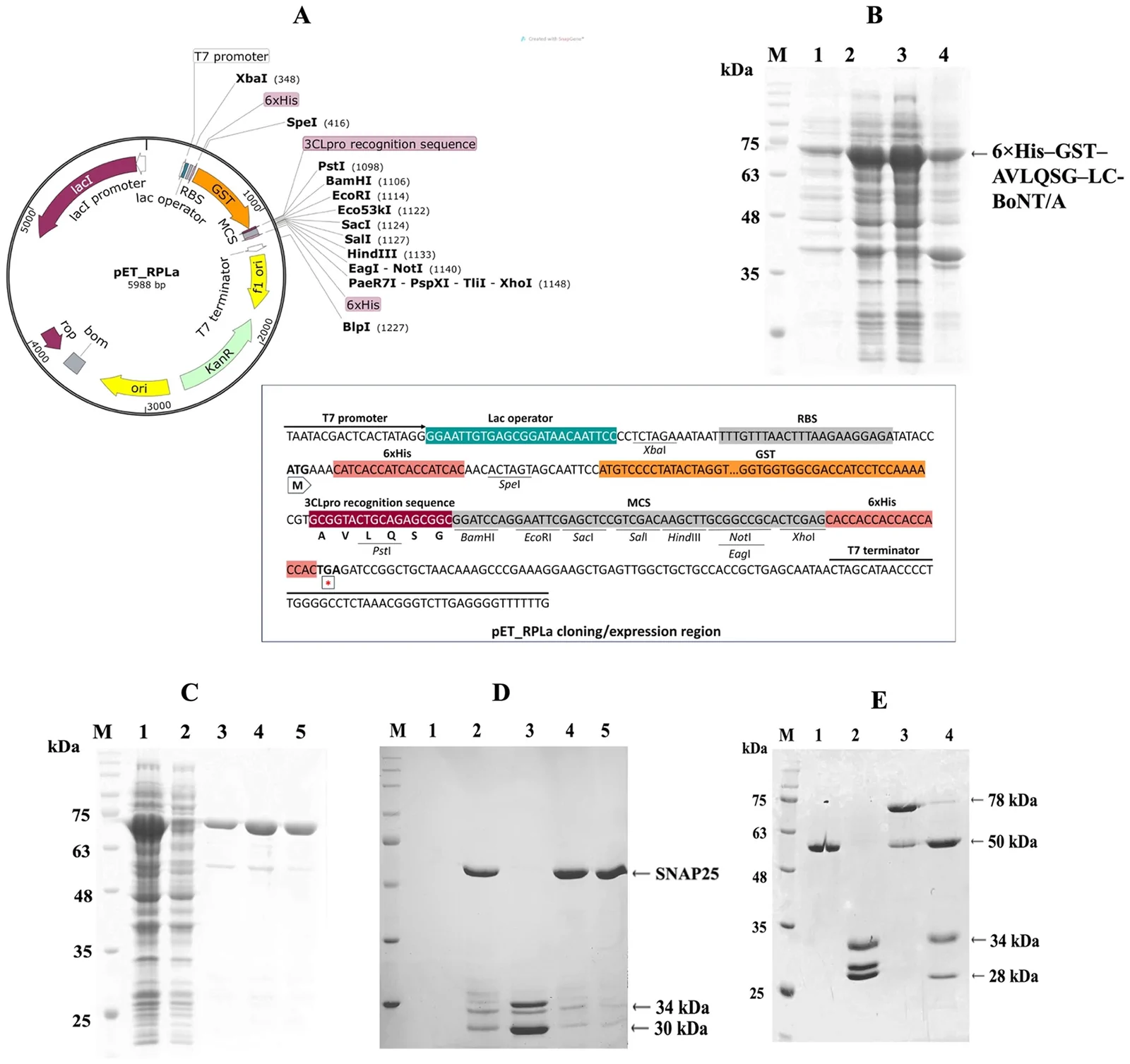
Schematic representation of the pET_RPLa vector and its application for expression of recombinant protein BoNT/A. **(A)** Schematic representation of the pET_RPLa vector showing the modular expression cassette (6 × His-GST-AVLQSG-MCS-6 × His) and key regulatory elements. **(B)** SDS–PAGE analysis of protein expression in *E. coli* BL21(DE3) harboring pET_RPLa-LC-BoNT/A. Molecular mass marker (lane M); the whole-cell lysates from non-induced and IPTG-induced cultures (lanes 1 and 2, respectively); soluble fraction (lane 3); insoluble fraction (lane 4). **(C)** SDS–PAGE analysis of Ni-Sepharose affinity purification of 6 × His-GST-AVLQSG-LC-BoNT/A. Molecular mass marker (lane M); crude lysate (lane 1); flow-through fraction (lane 2); elution fractions containing 6 × His-GST-AVLQSG-LC-BoNT/A (lanes 3-5). **(D)** SDS-PAGE analysis of SNAP25 cleavage assay. Molecular mass marker (lane M); 6 × His-GST-AVLQSG-LC-BoNT/A (lane 1); SNAP25 (lane 2); SNAP25 was cut with recombinant LC-BoNT/A (lane 3); SNAP25 was incubated with heat-inactivated recombinant LC-BoNT/A (lane 4); SNAP25 was incubated with recombinant LC-BoNT/A in the presence of EDTA (lane 5). **(E)** SDS-PAGE analysis of 3CLpro-mediated cleavage of recombinant LC-BoNT/A containing the AVLQ↓SG recognition sequence. Molecular mass marker (lane M); 6 × His-GST-AVLQSG-EYFP (lane 1); cleavage reaction of 6 × His-GST-AVLQSG-EYFP with 3CLpro (lane 2); 6 × His-GST-AVLQSG-LC-BoNT/A (lane 3); cleavage reaction of 6 × His-GST-AVLQSG-LC-BoNT/A with 3CLpro (lane 4).

To evaluate the functionality of this vector, the gene encoding the light chain of botulinum neurotoxin serotype A (LC-BoNT/A) was inserted into pET_RPLa to generate pET_RPLa-*LC-BoNT/A*. As shown in Fig 6B, the expression of the fusion protein 6 × His-GST-AVLQSG-LC-BoNT/A in *E. coli* BL21(DE3) harboring pET_RPLa-*LC-BoNT/A* resulted in a prominent band at approximately 78 kDa on SDS-PAGE (lane 2), consistent with the predicted molecular mass, and the recombinant protein was more predominantly detected in the soluble fraction (lane 3) than insoluble fraction (lane 4). 6 × His-GST-AVLQSG-LC-BoNT/A was purified to electrophoretic homogeneity by using Ni-Sepharose affinity chromatography (Fig 6C, lanes 3–5). We next tested whether the recombinant LC-BoNT/A obtained from our purification workflow remained enzymatically active by incubating it with SNAP25. Following co-incubation, the recombinant LC-BoNT/A efficiently cleaved SNAP25, producing two distinct fragments of approximately 34 and 30 kDa (Fig 6D, lane 3), consistent with the expected cleavage products. In contrast, no cleavage was detected when the enzyme was heat-inactivated or when the reaction was performed in the presence of EDTA (Fig 6D, lanes 4 and 5). These results demonstrate that the recombinant LC-BoNT/A retained its proteolytic activity following expression and purification.

To evaluate the applicability of 3CLpro for fusion-tag removal, purified 6 × His-GST-AVLQ↓SG-LC-BoNT/A (~78 kDa) was incubated with recombinant 3CLpro. In addition to the band corresponding to recombinant 3CLpro (~34 kDa), two major cleavage products of approximately 50 and 28 kDa were observed (Fig 6E, lane 4), corresponding to LC-BoNT/A and the 6 × His-GST-AVLQ fragment, respectively. In contrast, the intact fusion protein remained unchanged in the absence of 3CLpro (Fig 6E, lane 3). These results demonstrate that 3CLpro efficiently and specifically cleaved the engineered AVLQ↓SG recognition sequence, releasing LC-BoNT/A from the fusion partner.

Taken together, these results show that the pET_RPLa enabled successful expression of protein 6 × His-GST-AVLQSG-LC-BoNT/A in *E. coli* BL21(DE3), and that the target protein LC-BoNT/A was readily processed and purified by affinity chromatography in combination with 3CLpro digestion.

## Discussion

Proteases constitute one of the largest and most diverse enzyme families in nature. They are present in all living organisms and participate in a wide range of biological processes, including protein turnover, signal transduction, development, immunity, and host-pathogen interactions. Despite the enormous diversity of naturally occurring proteases, only a limited number possess sufficient sequence specificity to recognize and cleave a defined peptide motif with the precision required for recombinant fusion-tag removal [8]. In this study, we evaluated SARS-CoV-2 3CLpro as a sequence-specific protease for fusion-tag removal and further developed a modular expression vector incorporating a validated 3CLpro recognition sequence for recombinant protein production and subsequent proteolytic processing.

3CLpro specifically cleaved the 6 × His-GST-AVLQSG-EYFP substrate containing the canonical AVLQ↓SG recognition motif, generating the expected 6 × His-GST (~29 kDa) and EYFP (~27 kDa) fragments. In contrast, no cleavage was observed for the 6 × His-GST-TVRLQSG-EYFP variant, despite previous substrate-profiling studies identifying TVRLQ↓SGF as a highly favorable substrate sequence for SARS-CoV 3CLpro [22] and subsequent structural analyses demonstrating that substrate recognition is governed by interactions between residues surrounding the cleavage site and the corresponding substrate-binding pockets of the enzyme [15,23,24]. One possible explanation is that cleavage efficiency may depend on features beyond the recognition sequence, such as its structural context and accessibility within the fusion protein. These findings, therefore, emphasize the importance of experimentally validating cleavage motifs within the context of full-length recombinant protein intended for downstream applications.

A previous study [4] demonstrated the feasibility of using SARS-CoV-2 3CLpro for fusion-protein processing in *E. coli* through two *in vivo* strategies. In the first, 3CLpro and a GST fusion of the receptor-binding domain (RBD) of the SARS-CoV-2 spike protein were expressed from separate plasmids, allowing 3CLpro to cleave the fusion protein in the periplasm. In the second, GST, 3CLpro, and RBD were encoded within a single polyprotein, enabling autocatalytic release of 3CLpro followed by liberation of the RBD. Both approaches resulted in soluble RBD in the periplasm, although a portion of the protein remained insoluble [4]. The constructs were designed and evaluated specifically with RBD as the target protein. In contrast, the present study combines separately produced recombinant 3CLpro with a modular expression vector containing a validated 3CLpro recognition sequence, into which different target genes can be cloned for subsequent expression and proteolytic processing. Thus, the present study complements the previous *in vivo* approach by evaluating *in vitro* 3CLpro-mediated cleavage under purification-relevant conditions and across different cleavage formats.

Previous studies characterized the catalytic properties of coronavirus 3CL proteases under different reaction conditions, primarily using peptide-based or fluorogenic substrates [16,17]. The effects of pH and temperature observed in the present study were generally consistent with these previous findings, with higher activity at near-neutral pH and at 37°C [16,17]. Importantly, the present study evaluated 3CLpro-mediated cleavage using a full-length recombinant fusion protein under conditions directly relevant for fusion-tag removal workflows. 3CLpro remained active at 4°C and in the presence of affinity-elution reagents (glutathione, imidazole, and maltose), salts (NaCl, KCl, MgCl_2_, and MgSO_4_), stabilizers and detergents (glycerol, Triton X-100, and Tween 20), reducing agents (β-mercaptoethanol and DTT), as well as other protease inhibitors (EDTA and PMSF). These findings indicate that 3CLpro activity is retained under a range of conditions commonly encountered during recombinant protein purification. Activity at 4°C may be particularly useful for processing temperature-sensitive proteins, while tolerance to commonly used purification reagents may reduce the need for extensive buffer exchange before tag removal, thereby simplifying downstream processing.

The differential effects of the tested additives may be related to their distinct effects on protein structure and solubility. Urea and guanidine-HCl are chaotropic agents commonly used to solubilize aggregated proteins and inclusion bodies under denaturing conditions, whereas SDS is a strongly denaturing anionic detergent. Their inhibitory effects on 3CLpro-mediated cleavage are therefore consistent with disruption of the native protein conformation required for enzyme activity. In contrast, the nonionic detergents Tween 20 and Triton X-100 are generally milder toward protein structure and have been used to promote protein solubilization and refolding [25]. The enhanced cleavage observed in the presence of Tween 20 is also consistent with the modest increase in 3CLpro activity previously observed using a fluorogenic substrate [16]. ZnSO_4_ also inhibited cleavage, consistent with previous biochemical and structural evidence showing that Zn² ⁺ inhibits SARS-CoV-2 3CLpro through coordination with the catalytic dyad at the active site [26].

The versatility of 3CLpro was further demonstrated by its application in multiple cleavage formats, including in-solution, on-column, and during-dialysis reactions. Such flexibility is a desirable characteristic for proteases used in affinity-tag removal, as compatibility with diverse purification workflows can simplify downstream processing and minimize additional handling steps. In-solution cleavage provides a straightforward approach after protein purification, whereas on-column cleavage can combine proteolytic processing with affinity-based separation of the cleaved target protein. Dialysis-coupled cleavage allows proteolysis and buffer exchange to proceed concurrently. Together, these results show that 3CLpro-mediated processing can be adapted to different downstream purification strategies rather than being restricted to a single cleavage format.

From a practical perspective, 3CLpro has several features that support its potential use for fusion-tag removal. TEV and HRV 3C proteases are well established and commercially available [5,8]. A previous comparative study reported an approximately 10-fold higher k_cat_ and 13-fold higher catalytic efficiency (k_cat_/K_m_) for SARS-CoV-2 3CLpro than for recombinant TEV protease using similarly designed fluorogenic peptide substrates [4]. The present results further show that 3CLpro remains active at low temperature and is compatible with a range of reagents commonly encountered during protein purification, providing flexibility under different protein-processing conditions.

To further expand the utility of 3CLpro in recombinant protein production, we developed the modular expression vector pET_RPLa incorporating our validated 3CLpro recognition sequence. The functionality of this vector was demonstrated using the light chain of botulinum neurotoxin type A (LC-BoNT/A) as a model protein. The resulting fusion protein was expressed predominantly in soluble form, purified by affinity chromatography, retained SNAP25-cleaving activity, and was efficiently processed by recombinant 3CLpro. Unlike the EYFP-based construct used to characterize 3CLpro-mediated cleavage, pET_RPLa was designed as a modular expression platform to facilitate recombinant protein production and subsequent tag removal.

The biochemical characterization was conducted primarily using the 6 × His-GST-AVLQSG-EYFP substrate, while the modular expression platform was further validated with LC-BoNT/A. Although these results demonstrate the feasibility of 3CLpro-mediated processing in two different protein contexts, broader validation with proteins of diverse structural and biochemical properties is needed to establish the general applicability of this approach. Determining kinetic parameters for cleavage of full-length fusion proteins, and benchmarking 3CLpro directly against established tag-removal proteases such as TEV or HRV 3C under identical conditions, are important priorities for future work. The economic feasibility of scaling this approach also remains to be evaluated. Future studies addressing these aspects will help define the range of applications and practical advantages of 3CLpro-mediated fusion-tag removal.

## Conclusion

In conclusion, the present study demonstrates the feasibility of using recombinant SARS-CoV-2 3CLpro as an effective site-specific protease for fusion-tag removal. Its sequence-dependent cleavage, activity under purification-relevant conditions, and compatibility with different cleavage formats support its potential integration into recombinant protein purification workflows. The pET_RPLa expression vector further provides a platform for combining recombinant protein expression with subsequent 3CLpro-mediated tag removal. Together, these findings establish SARS-CoV-2 3CLpro as a promising addition to the repertoire of proteases available for recombinant protein processing and purification.

## Supporting information

S1 File Available original images underlying the gel and blot results. This file contains the original uncropped and minimally adjusted images underlying the gel and Western blot results presented in the manuscript.
(PDF)

S2 File Verification of the pET_RPLa vector by PCR and Sanger sequencing. This file contains PCR verification data, Sanger sequencing chromatograms, and sequence alignments confirming the correct construction of the pET_RPLa vector.
(PDF)

S3 File ImageJ densitometric data underlying Figs 3 and 4. Band-intensity measurements and calculated cleavage values for the experimental conditions examined.
(PDF)

## Abbreviations

3CLpro: SARS-CoV-2 3C-like protease
EYFP: enhanced yellow fluorescent protein
GST: glutathione S-transferase
HRV: Human rhinovirus
LC-BoNT/A: light chain of botulinum neurotoxin serotype A
MBP: maltose-binding protein
PCR: polymerase chain reaction
SDS-PAGE: sodium dodecyl sulfate-polyacrylamide gel electrophoresis
SNAP25: synaptosome-associated protein 25
TEV: tobacco etch virus

## References

[1] Costa S, Almeida A, Castro A, Domingues L. Fusion tags for protein solubility, purification and immunogenicity in *Escherichia coli*: The novel Fh8 system. Front Microbiol. 2014;5:63. doi: 10.3389/fmicb.2014.00063 24600443 PMC3928792

[2] Rosano GL, Ceccarelli EA. Recombinant protein expression in *Escherichia coli*: Advances and challenges. Front Microbiol. 2014;5:172. doi: 10.3389/fmicb.2014.00172 24860555 PMC4029002

[3] Terpe K. Overview of tag protein fusions: From molecular and biochemical fundamentals to commercial systems. Appl Microbiol Biotechnol. 2003;60(5):523–33. doi: 10.1007/s00253-002-1158-6 12536251

[4] Belenkaya SV, Merkuleva IA, Yarovaya OI, Chirkova VY, Sharlaeva EA, Shanshin DV, et al. The main protease 3CLpro of the SARS-CoV-2 virus: How to turn an enemy into a helper. Front Bioeng Biotechnol. 2023;11:1187761. doi: 10.3389/fbioe.2023.1187761 37456729 PMC10345205

[5] Ullah R, Shah MA, Tufail S, Ismat F, Imran M, Iqbal M, et al. Activity of the human rhinovirus 3C protease studied in various buffers, additives and detergents solutions for recombinant protein production. PLoS One. 2016;11(4):e0153436. doi: 10.1371/journal.pone.0153436 27093053 PMC4836831

[6] Kühnel B, Alcantara J, Boothe J, van Rooijen G, Moloney M. Precise and efficient cleavage of recombinant fusion proteins using mammalian aspartic proteases. Protein Eng. 2003;16(10):777–83. doi: 10.1093/protein/gzg091 14600208

[7] Jenny RJ, Mann KG, Lundblad RL. A critical review of the methods for cleavage of fusion proteins with thrombin and factor Xa. Protein Expr Purif. 2003;31(1):1–11. doi: 10.1016/s1046-5928(03)00168-2 12963335

[8] Waugh DS. An overview of enzymatic reagents for the removal of affinity tags. Protein Expr Purif. 2011;80(2):283–93. doi: 10.1016/j.pep.2011.08.005 21871965 PMC3195948

[9] Shams R, Regan L. A new highly specific and soluble protease for precise removal of N‑terminal purification tags. ACS Omega. 2025;10(28):30354–64. doi: 10.1021/acsomega.5c01764 40727744 PMC12290709

[10] Fan X, Li X, Zhou Y, Mei M, Liu P, Zhao J, et al. Quantitative analysis of the substrate specificity of human rhinovirus 3C protease and exploration of its substrate recognition mechanisms. ACS Chem Biol. 2020;15(1):63–73. doi: 10.1021/acschembio.9b00539 31613083

[11] Zhang L, Lin D, Sun X, Curth U, Drosten C, Sauerhering L, et al. Crystal structure of SARS-CoV-2 main protease provides a basis for design of improved α-ketoamide inhibitors. Science. 2020;368(6489):409–12. doi: 10.1126/science.abb3405 32198291 PMC7164518

[12] Lee J, Kenward C, Worrall LJ, Vuckovic M, Gentile F, Ton A-T, et al. X-ray crystallographic characterization of the SARS-CoV-2 main protease polyprotein cleavage sites essential for viral processing and maturation. Nat Commun. 2022;13(1):5196. doi: 10.1038/s41467-022-32854-4 36057636 PMC9440467

[13] Tran T-HT, Nguyen T-D, Phan N-N, Ngo HT, Nguyen Do P-L, Le P-A, et al. A magnetic bead-based fluorescent substrate for sensitive assay of SARS-CoV-2 3C-like protease activity. Protein Expr Purif. 2025;236:106811. doi: 10.1016/j.pep.2025.106811 40915590

[14] Aniana A, Nashed NT, Ghirlando R, Bhandari D, Kovalevsky A, Louis JM. Cleavage at the nsp5-nsp6 site of SARS-CoV-2 main protease intermediate precursor is faster from a monomer than a dimer form. J Biol Chem. 2026;302(5):111395. doi: 10.1016/j.jbc.2026.111395 41866037 PMC13125995

[15] Kenward C, Vuckovic M, Paetzel M, Strynadka NCJ. Kinetic comparison of all eleven viral polyprotein cleavage site processing events by SARS-CoV-2 main protease using a linked protein FRET platform. J Biol Chem. 2024;300(6):107367. doi: 10.1016/j.jbc.2024.107367 38750796 PMC11209022

[16] Dražić T, Kühl N, Leuthold MM, Behnam MAM, Klein CD. Efficiency improvements and discovery of new substrates for a SARS-CoV-2 main protease FRET assay. SLAS Discov. 2021;26(9):1189–99. doi: 10.1177/24725552211020681 34151620 PMC8458682

[17] Nguyen H-LT, Nguyen N-QT, Le T-T, Pham X-DT, Pham H-L, Le H-NT, et al. Improved expression and purification of highly-active 3 chymotrypsin-like protease from SARS-CoV-2. Protein Expr Purif. 2024;215:106414. doi: 10.1016/j.pep.2023.106414 38072143

[18] Kuo C-J, Shih Y-P, Kan D, Liang P-H. Engineering a novel endopeptidase based on SARS 3CL(pro). Biotechniques. 2009;47(6):1029–32. doi: 10.2144/000113303 20041855 PMC7081961

[19] Bradford MM. A rapid and sensitive method for the quantitation of microgram quantities of protein utilizing the principle of protein-dye binding. Anal Biochem. 1976;72:248–54. doi: 10.1016/0003-2697(76)90527-3 942051

[20] Regan AD, Shraybman R, Cohen RD, Whittaker GR. Differential role for low pH and cathepsin-mediated cleavage of the viral spike protein during entry of serotype II feline coronaviruses. Vet Microbiol. 2008;132(3–4):235–48. doi: 10.1016/j.vetmic.2008.05.019 18606506 PMC2588466

[21] Luong HT, Kieu MB, Phi HT, Nguyen NH, Luu QM, Vu CT, et al. Nanobody-functionalized magnetic beads for enrichment of foodborne botulinum toxin type A light chain. J Microbiol Biotechnol. 2025;35:e2507042. doi: 10.4014/jmb.2507.07042 41084210 PMC12536258

[22] Chuck C-P, Chong L-T, Chen C, Chow H-F, Wan DC-C, Wong K-B. Profiling of substrate specificity of SARS-CoV 3CL. PLoS One. 2010;5(10):e13197. doi: 10.1371/journal.pone.0013197 20949131 PMC2950840

[23] Miczi M, Golda M, Kunkli B, Nagy T, Tőzsér J, Mótyán JA. Identification of host cellular protein substrates of SARS-COV-2 main protease. Int J Mol Sci. 2020;21(24):9523. doi: 10.3390/ijms21249523 33333742 PMC7765187

[24] Cesar Ramos de Jesus H, Solis N, Machado Y, Pablos I, Bell PA, Kappelhoff R, et al. Optimization of quenched fluorescent peptide substrates of SARS-CoV-2 3CLpro main protease (Mpro) from proteomic identification of P6-P6’ active site specificity. J Virol. 2024;98(6):e0004924. doi: 10.1128/jvi.00049-24 38742901 PMC11237654

[25] Lee S-H, Carpenter JF, Chang BS, Randolph TW, Kim Y-S. Effects of solutes on solubilization and refolding of proteins from inclusion bodies with high hydrostatic pressure. Protein Sci. 2006;15(2):304–13. doi: 10.1110/ps.051813506 16385003 PMC2242456

[26] Panchariya L, Khan WA, Kuila S, Sonkar K, Sahoo S, Ghoshal A, et al. Zinc^2+^ ion inhibits SARS-CoV-2 main protease and viral replication in vitro. Chem Commun (Camb). 2021;57(78):10083–6. doi: 10.1039/d1cc03563k 34514483

