# Supplementary material for: SARS-CoV-2 3CLpro as an efficient endopeptidase for fusion tag removal in recombinant protein production
Source: PLoS One. 2026 Nov 11;21(11):e0362685. doi: 10.1371/journal.pone.0362685 (PMC13754225; doi:10.1371/journal.pone.0362685)
Supplement: Supplementary file 1 [file pone.0362685.s001.pdf]

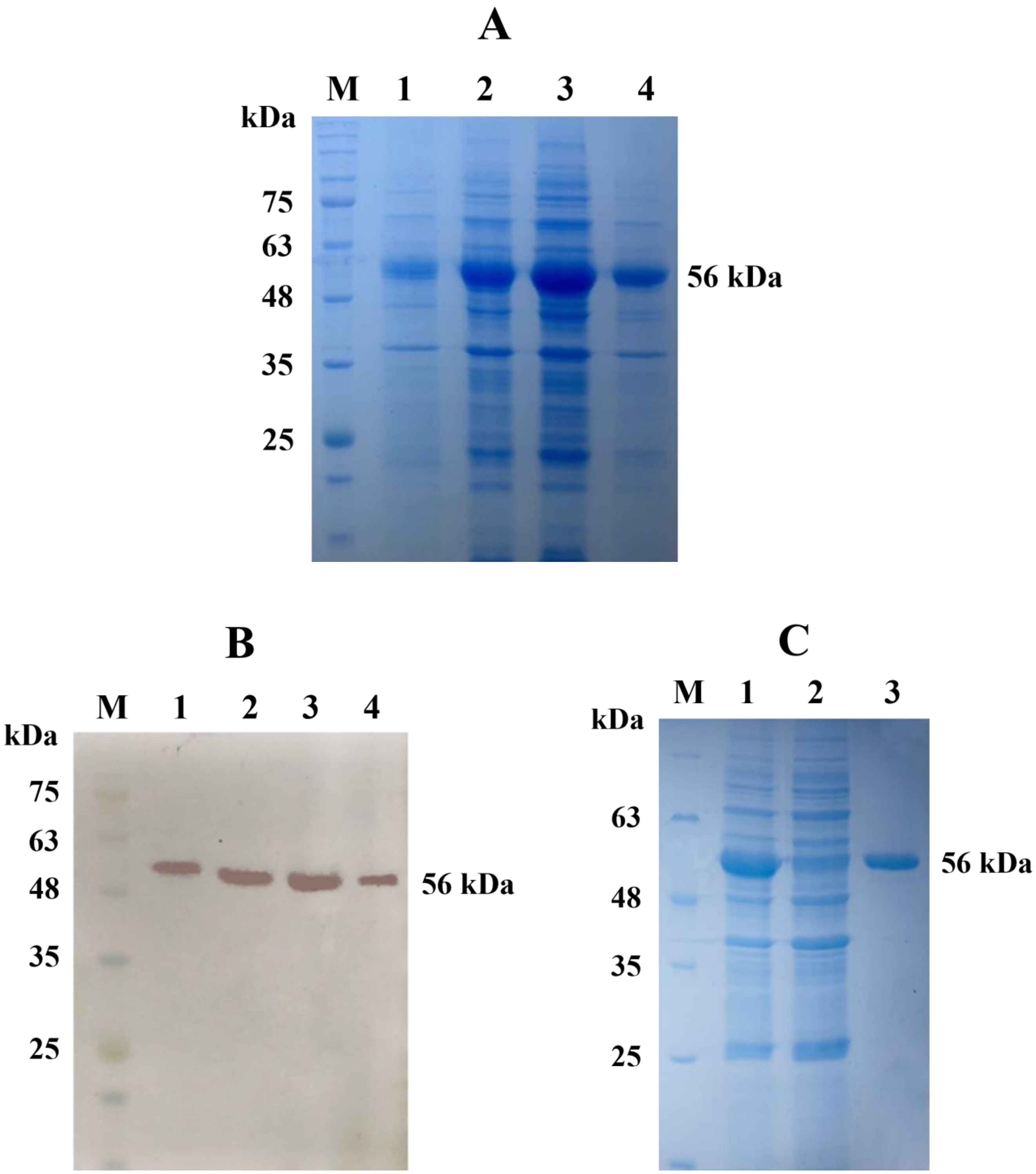

Fig 1. Expression and purification of 6×His-GST-AVLQSG-EYFP protein

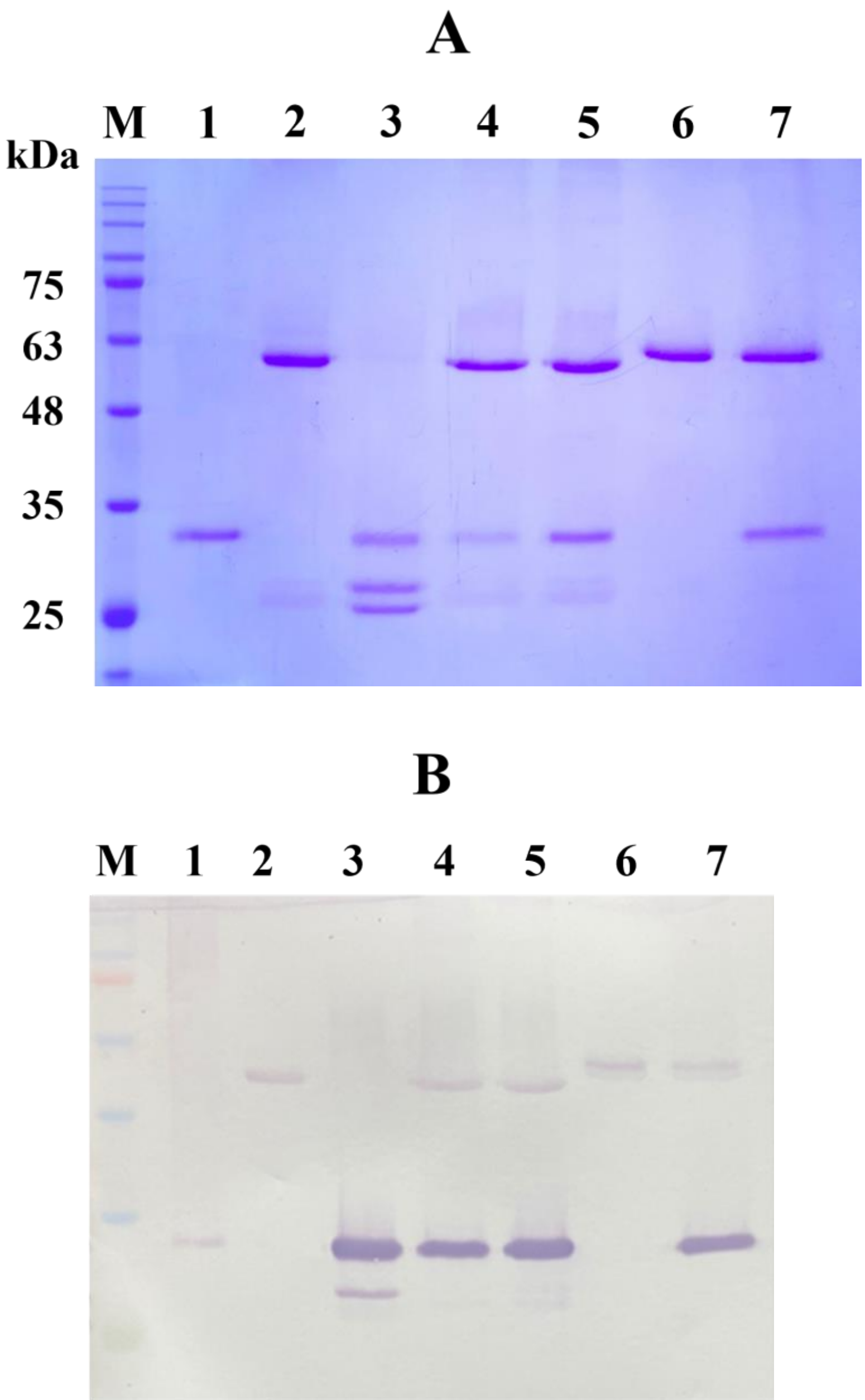
A
M 1 2 3 4 5 6 7
kDa
75
63
48
35
25
B
M 1 2 3 4 5 6 7

C

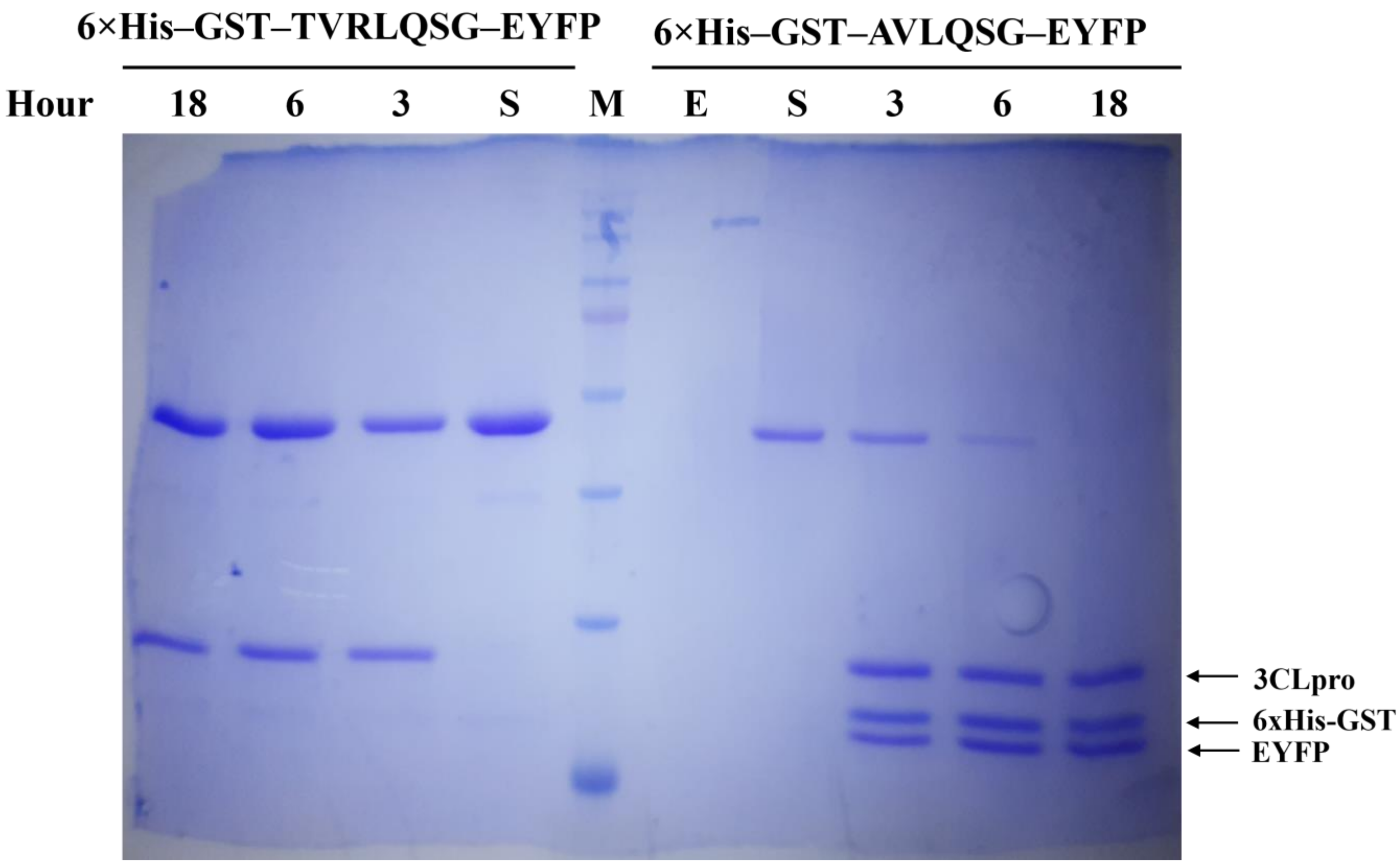

Fig 2. Specific cleavage of fusion substrates by 3CLpro and sequence-dependent substrate recognition

A

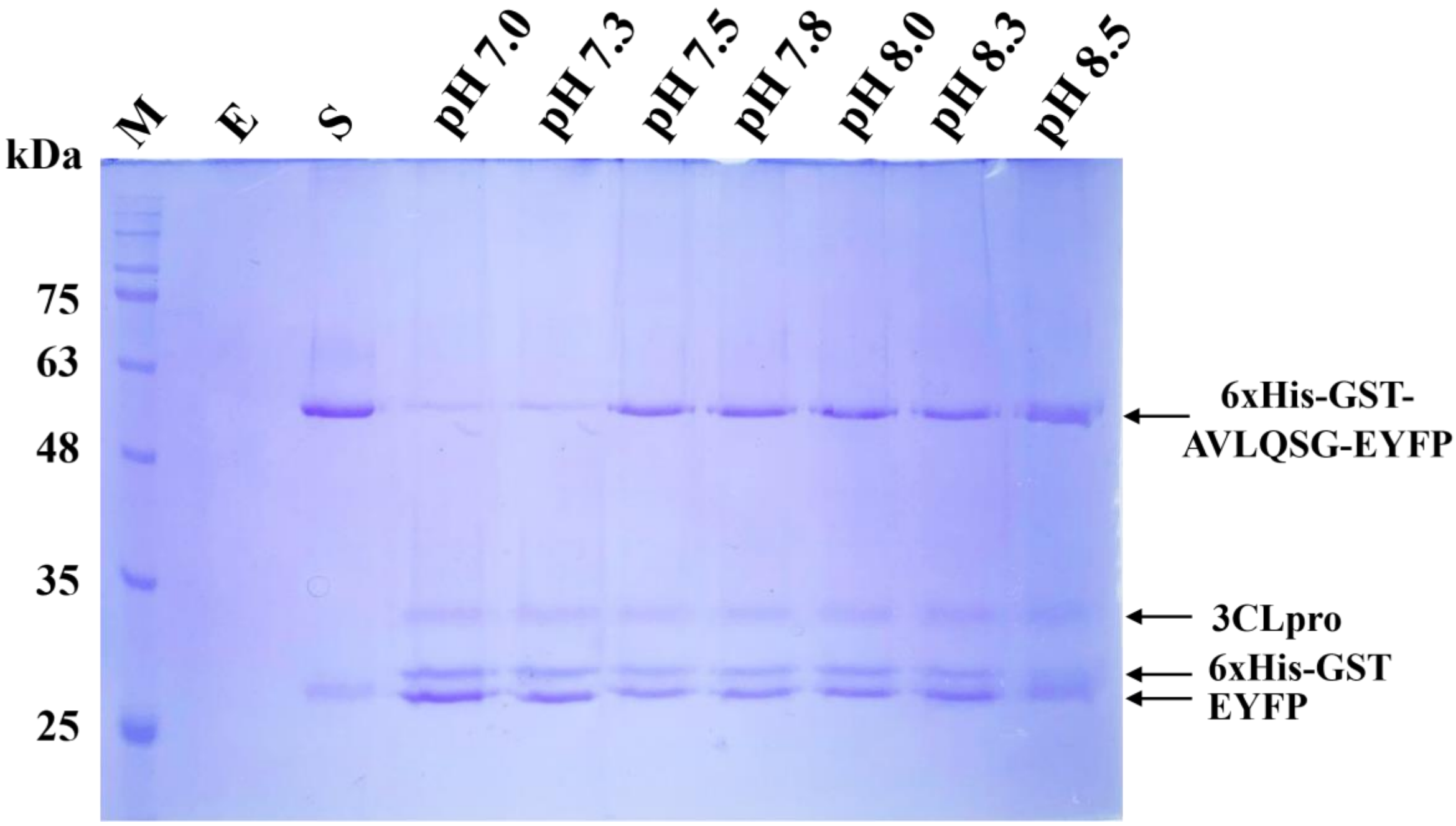
M
E
S
pH 7.0
pH 7.3
pH 7.5
pH 7.8
pH 8.0
pH 8.3
pH 8.5
kDa
75
63
48
35
25
6xHis-GST-AVLQSG-EYFP
3CLpro
6xHis-GST
EYFP

B

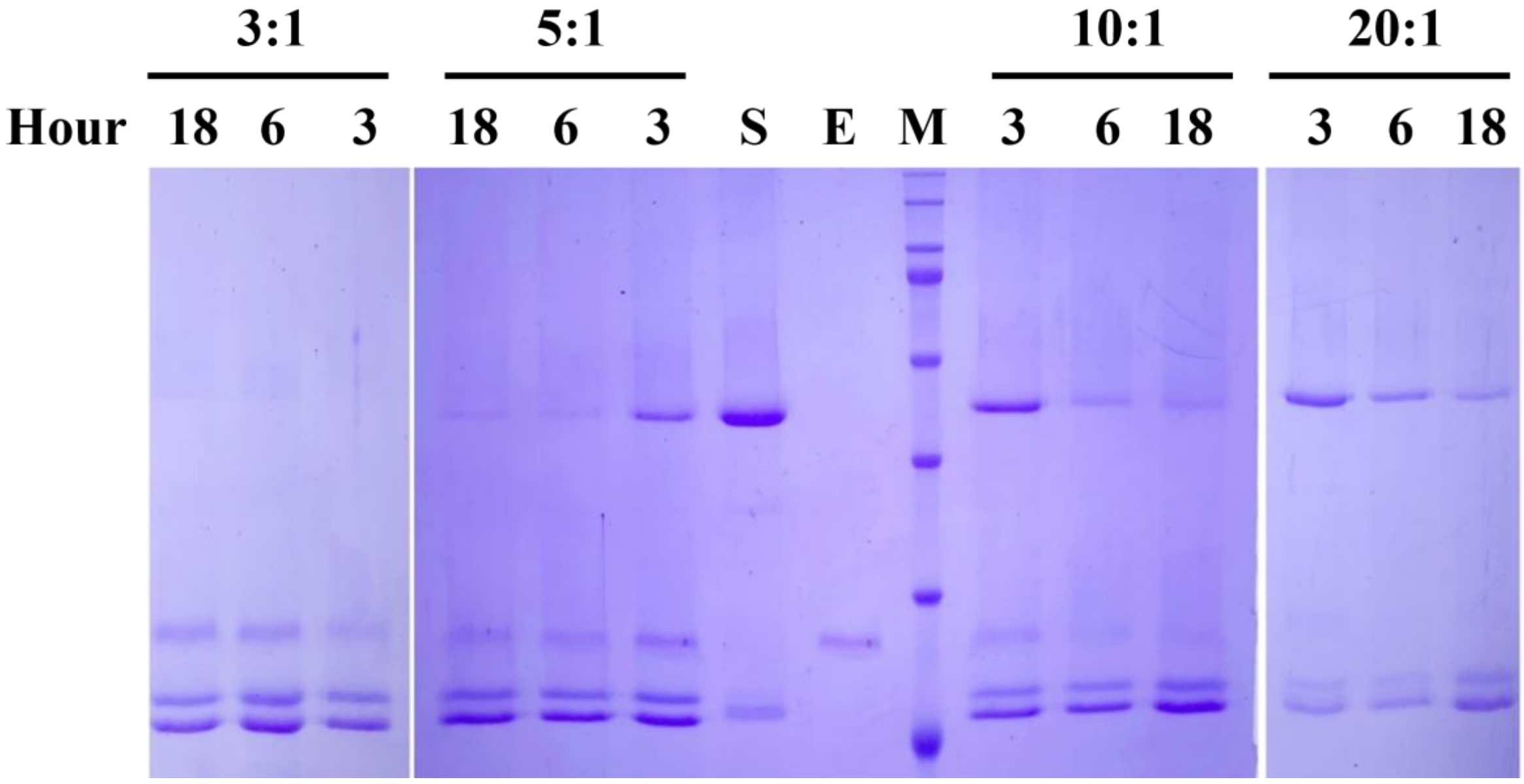
3:1
5:1
10:1
20:1
Hour
18
6
3
18
6
3
S
E
M
3
6
18
3
6
18

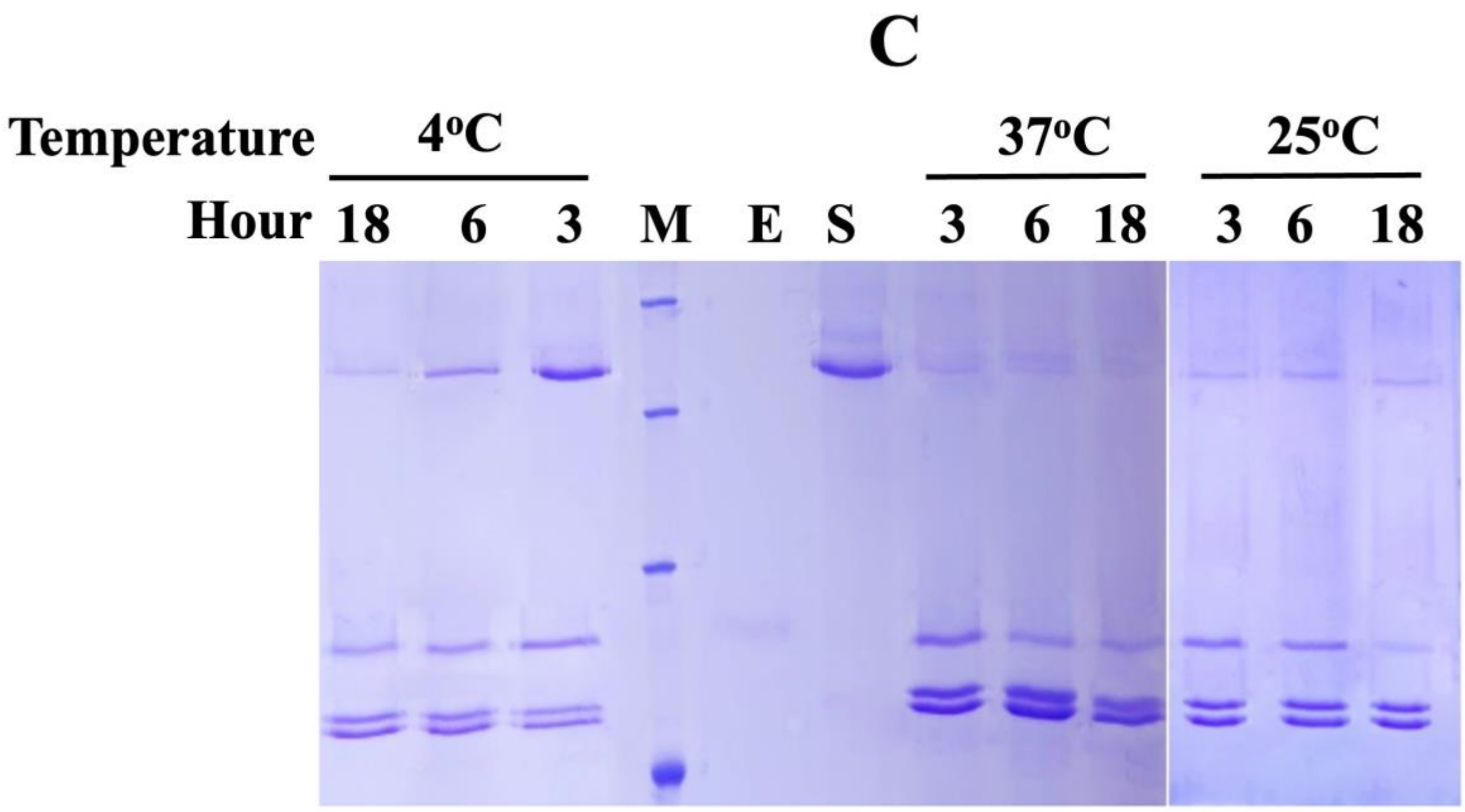

Fig 3. Effects of reaction conditions on 3CLpro-mediated cleavage of the recombinant substrate

Affinity chromatography eluents

Imidazole (mM)

x M S E 0 10 50 100 200

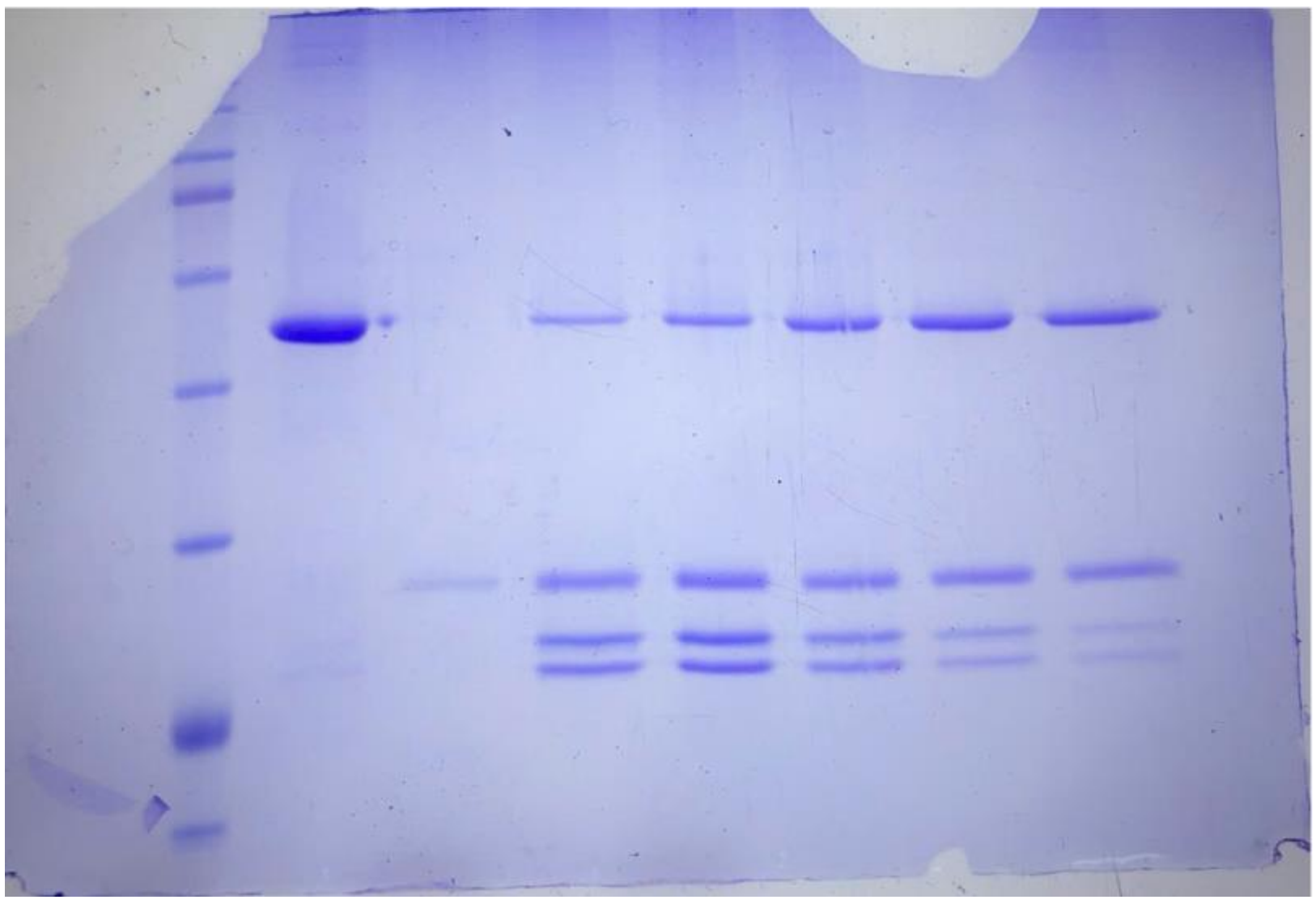

Reduced glutathione (mM)

x M S E 0 0.1 1.0 5.0 10 x

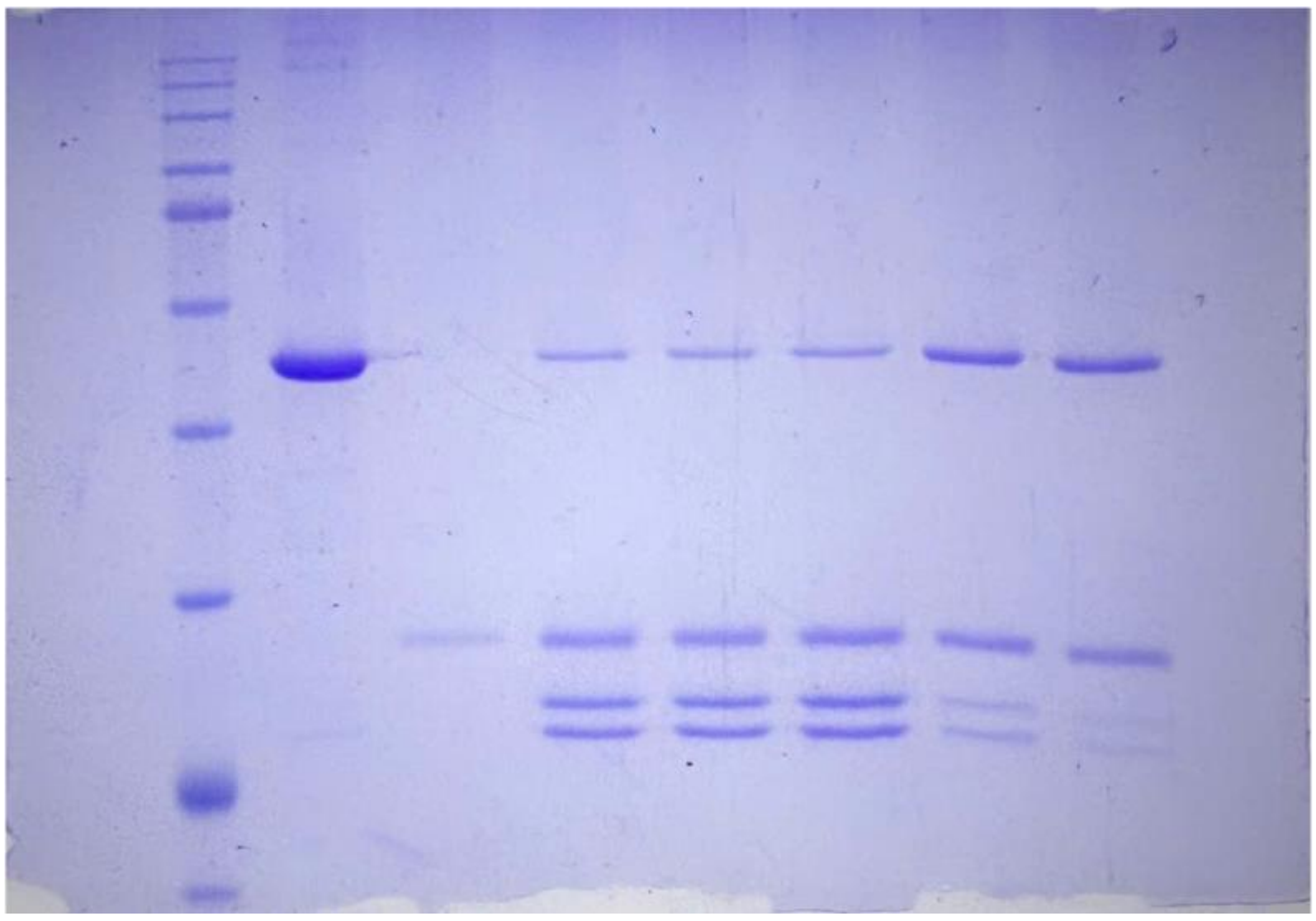

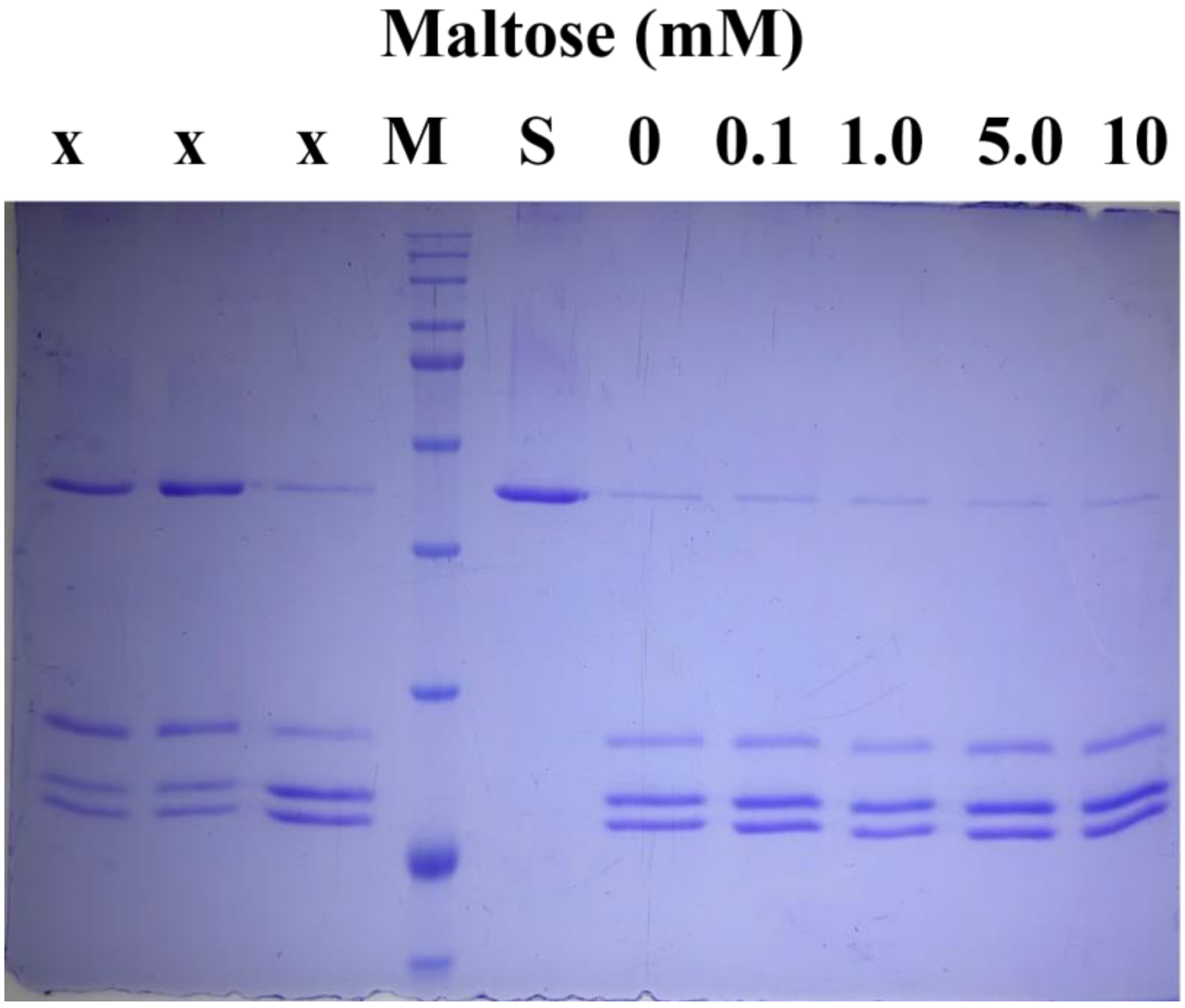
Maltose (mM)
x x x M S 0 0.1 1.0 5.0 10

Salts

NaCl (mM)

x M S 0 150 300 600 800 x

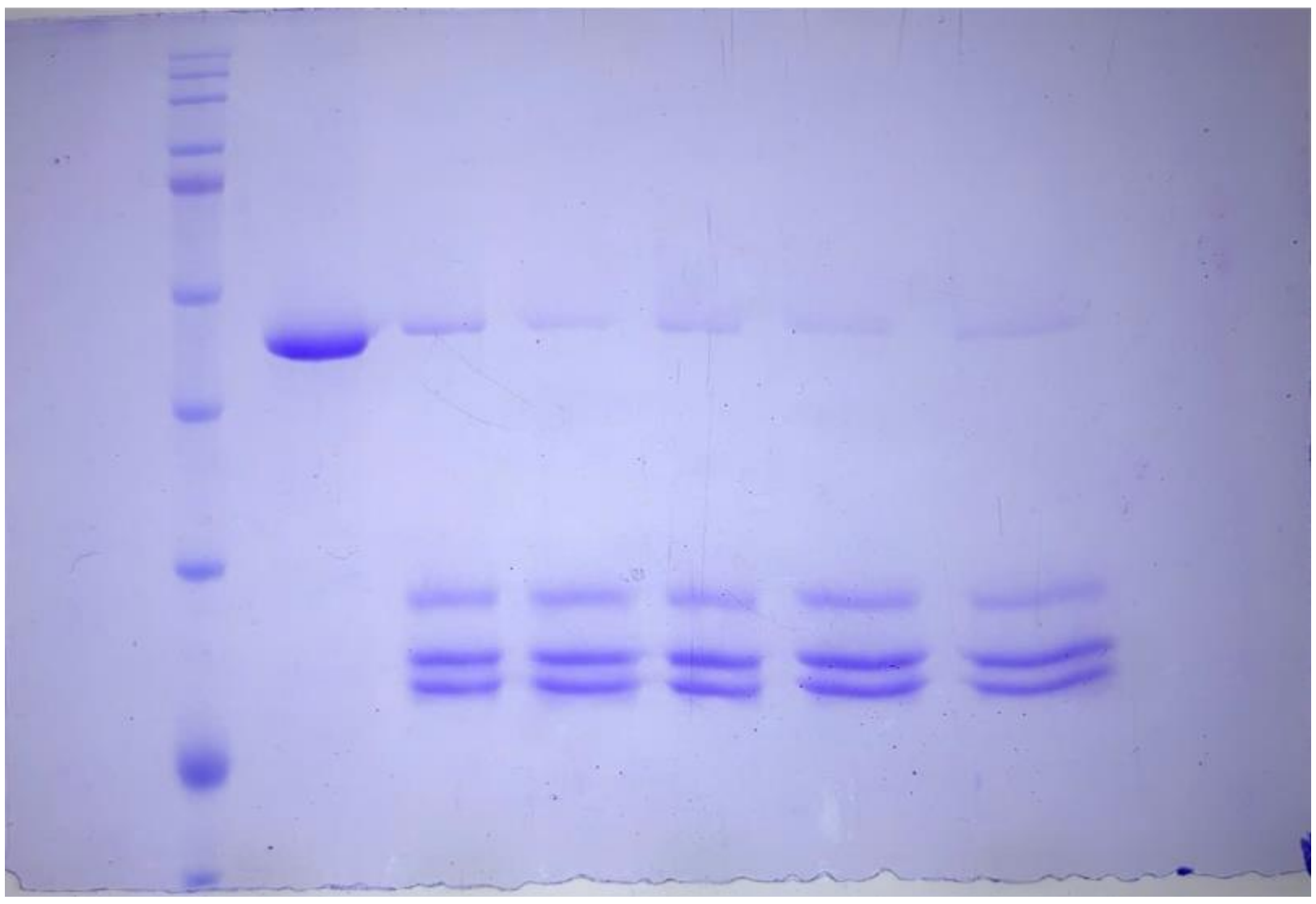

KCl (mM)

x x M S 0 150 300 400 600 x

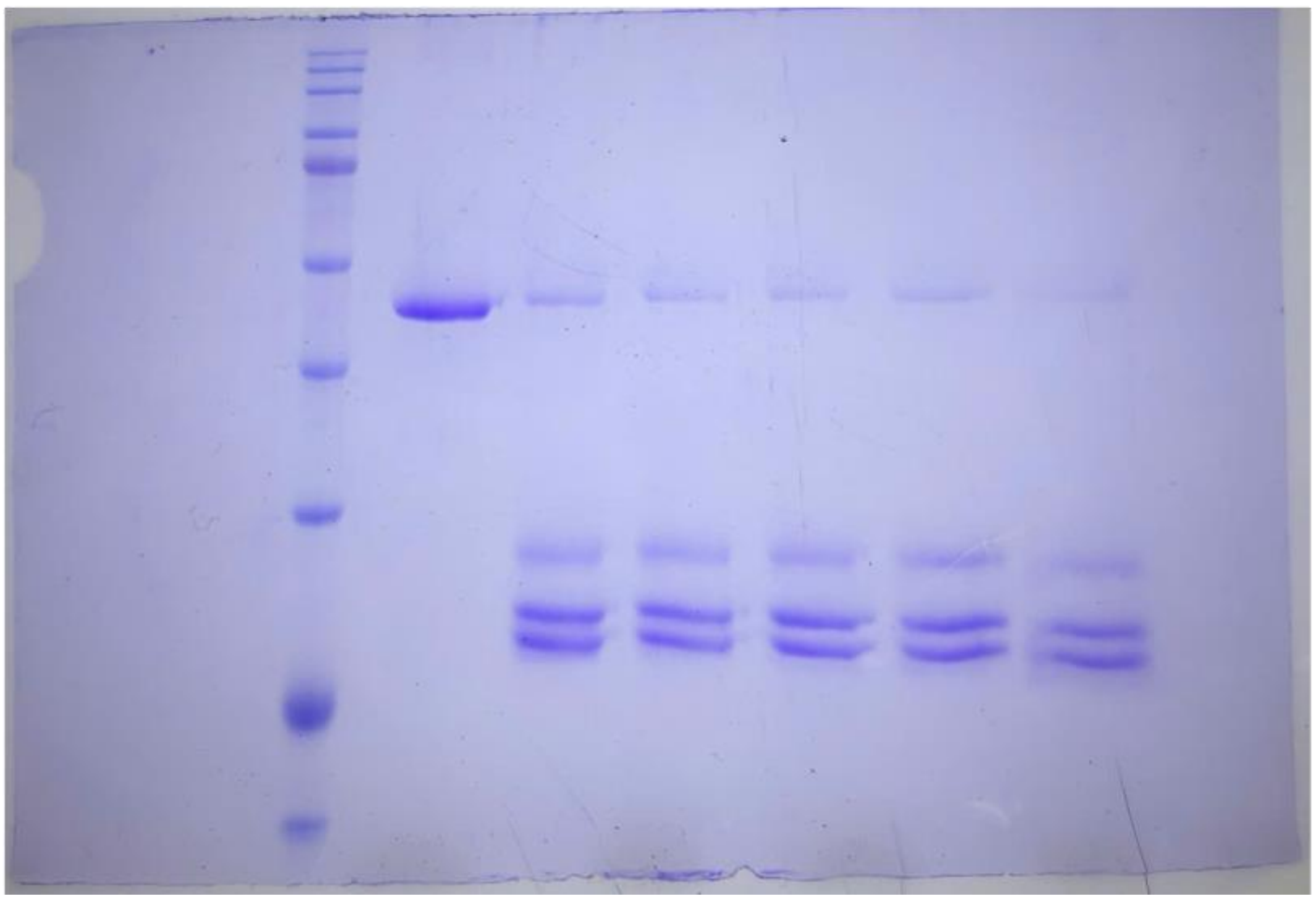

$CaCl_2$ (mM)

M S 0 1 2 5 10

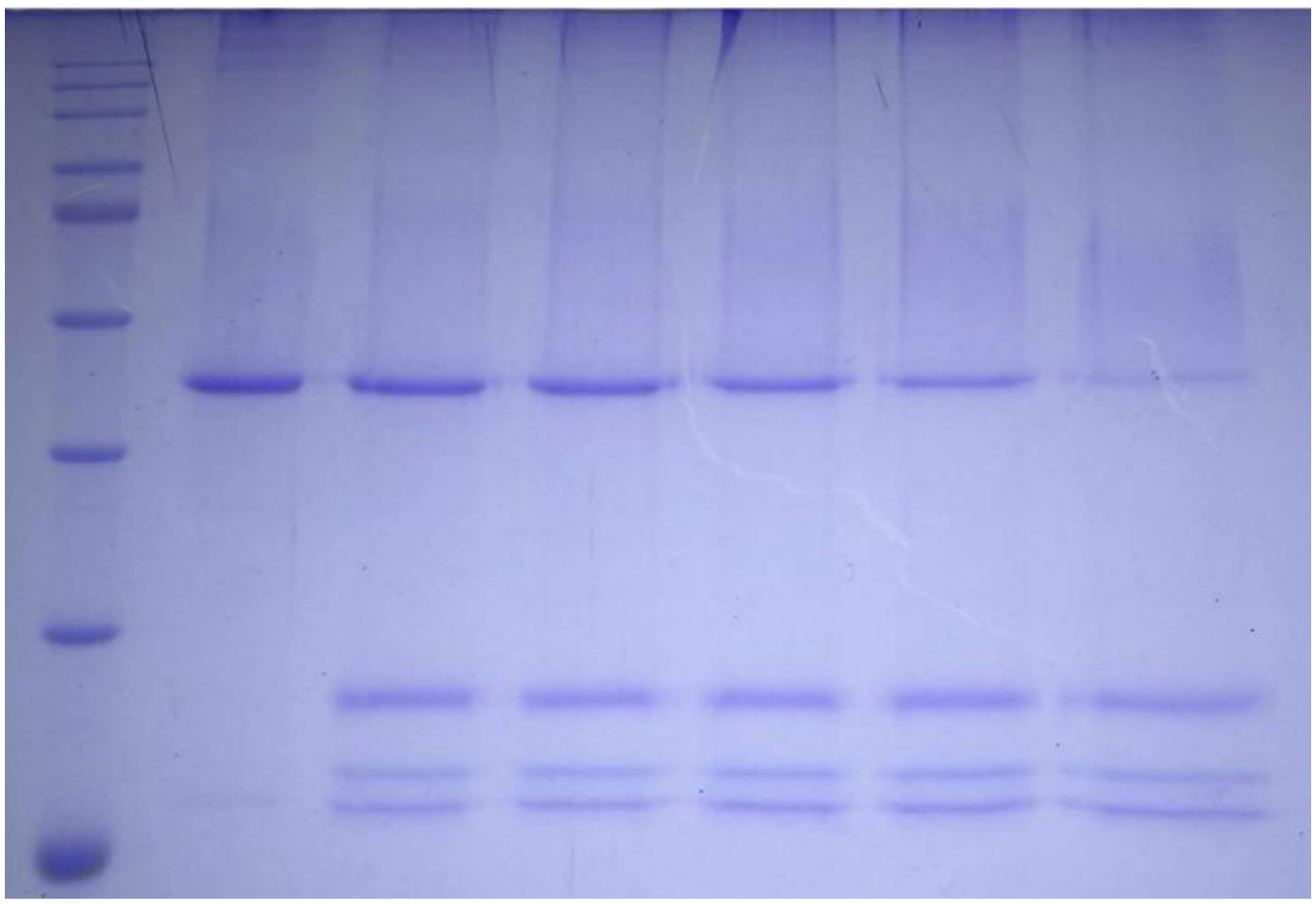

$MgCl_2$ (mM)

x M S 0 1 5 10 25 x x

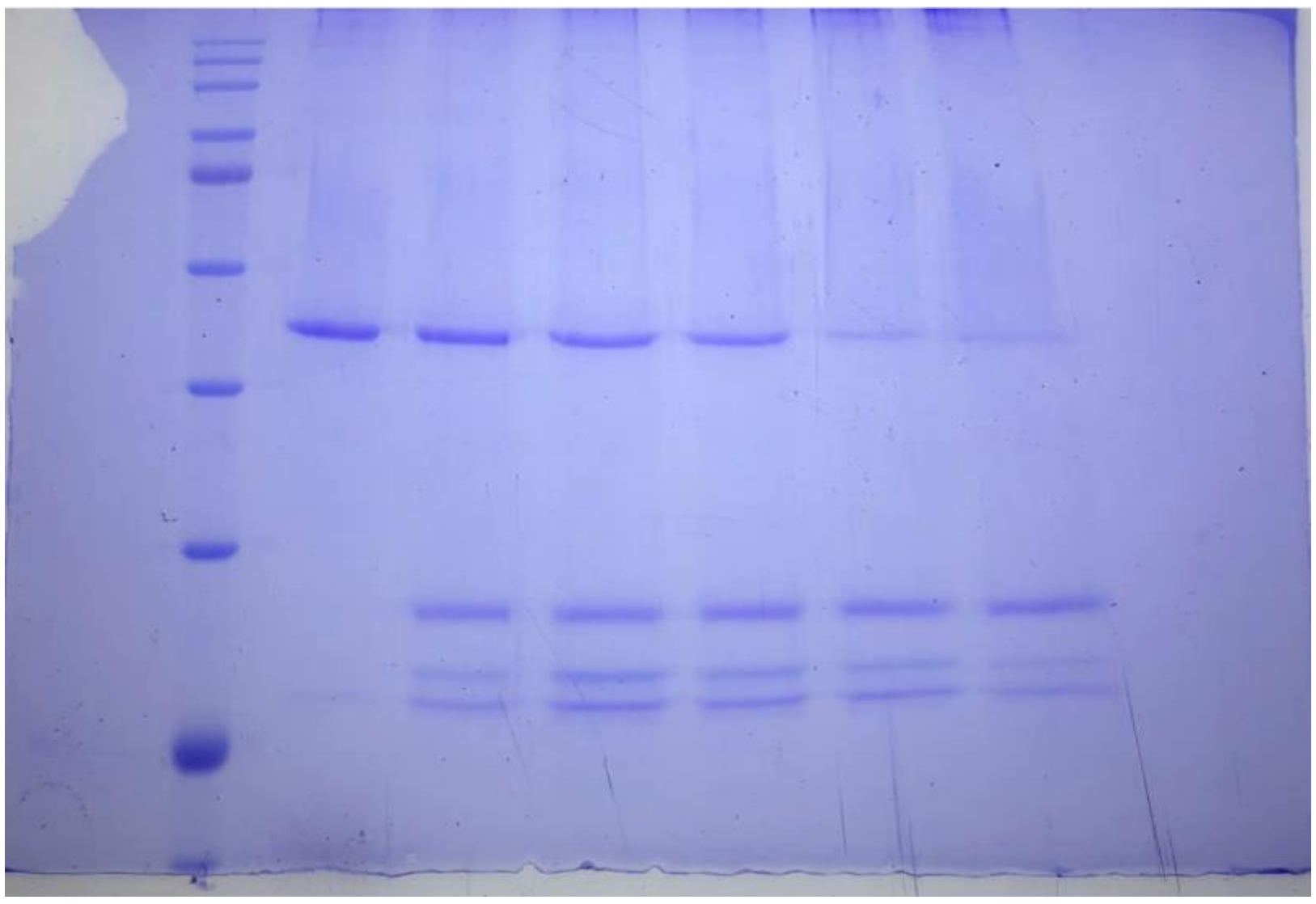

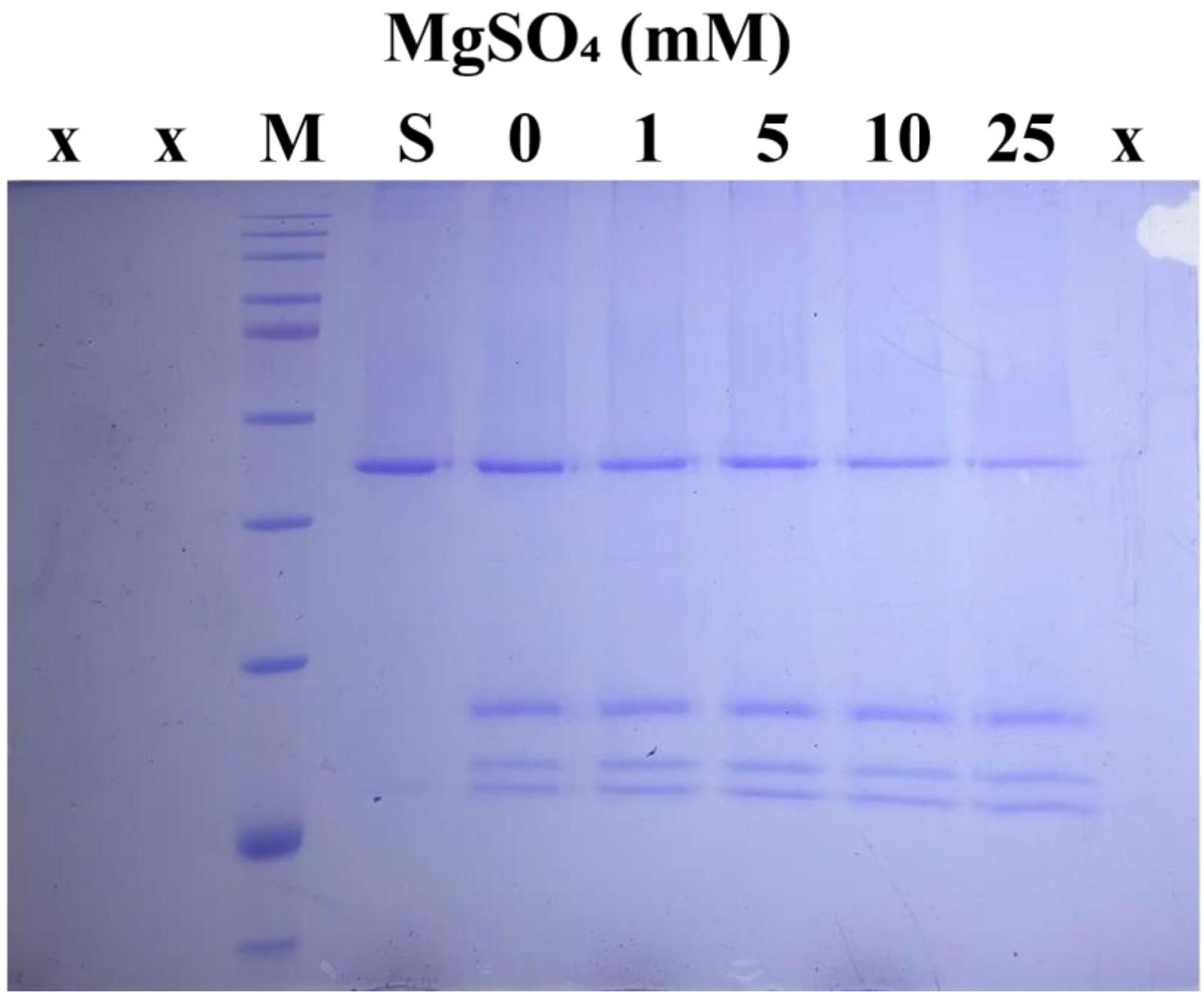
MgSO4 (mM)
x x M S 0 1 5 10 25 x

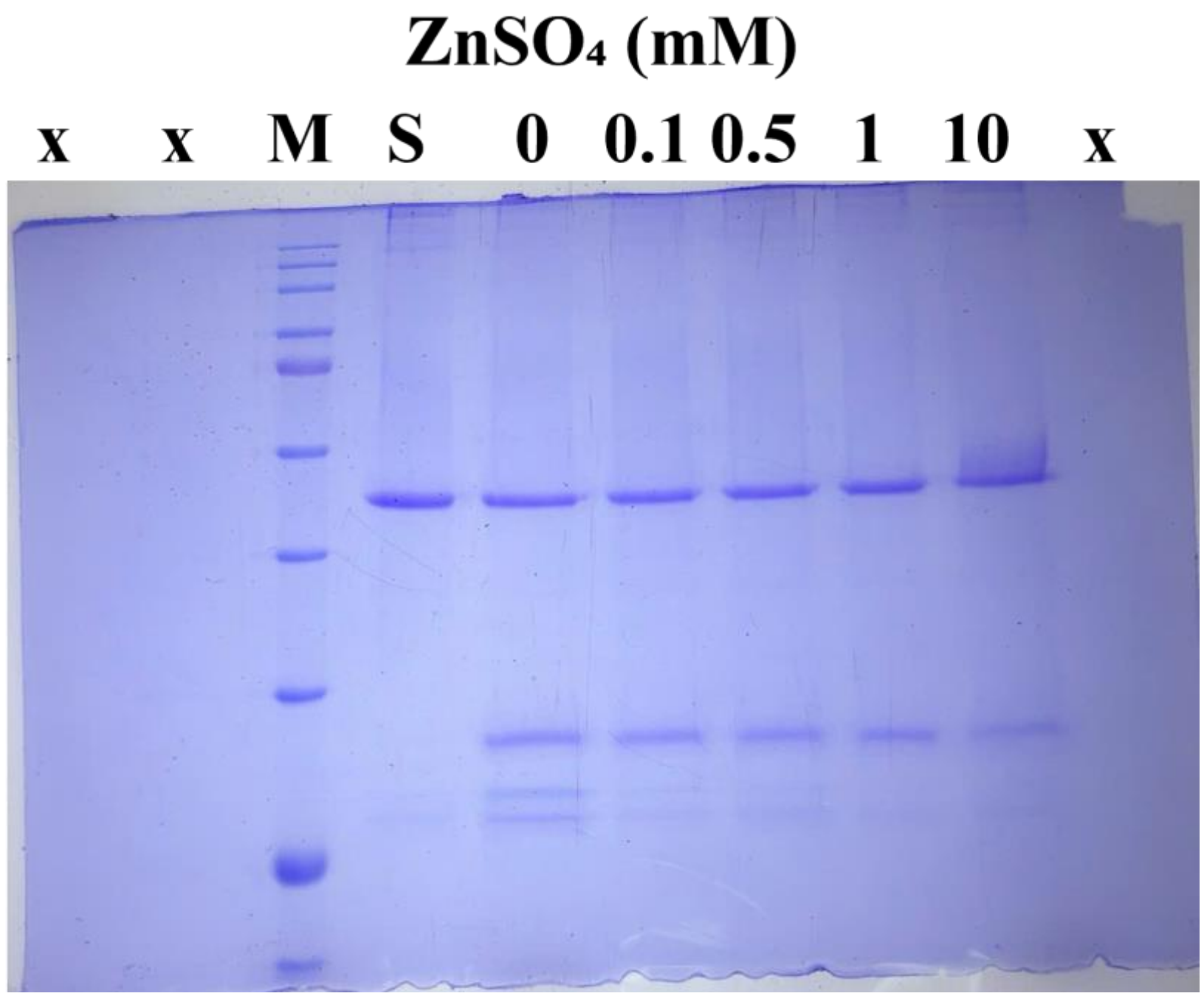
ZnSO4 (mM)
x x M S 0 0.1 0.5 1 10 x

Stabilizers/detergents & chelator/inhibitor

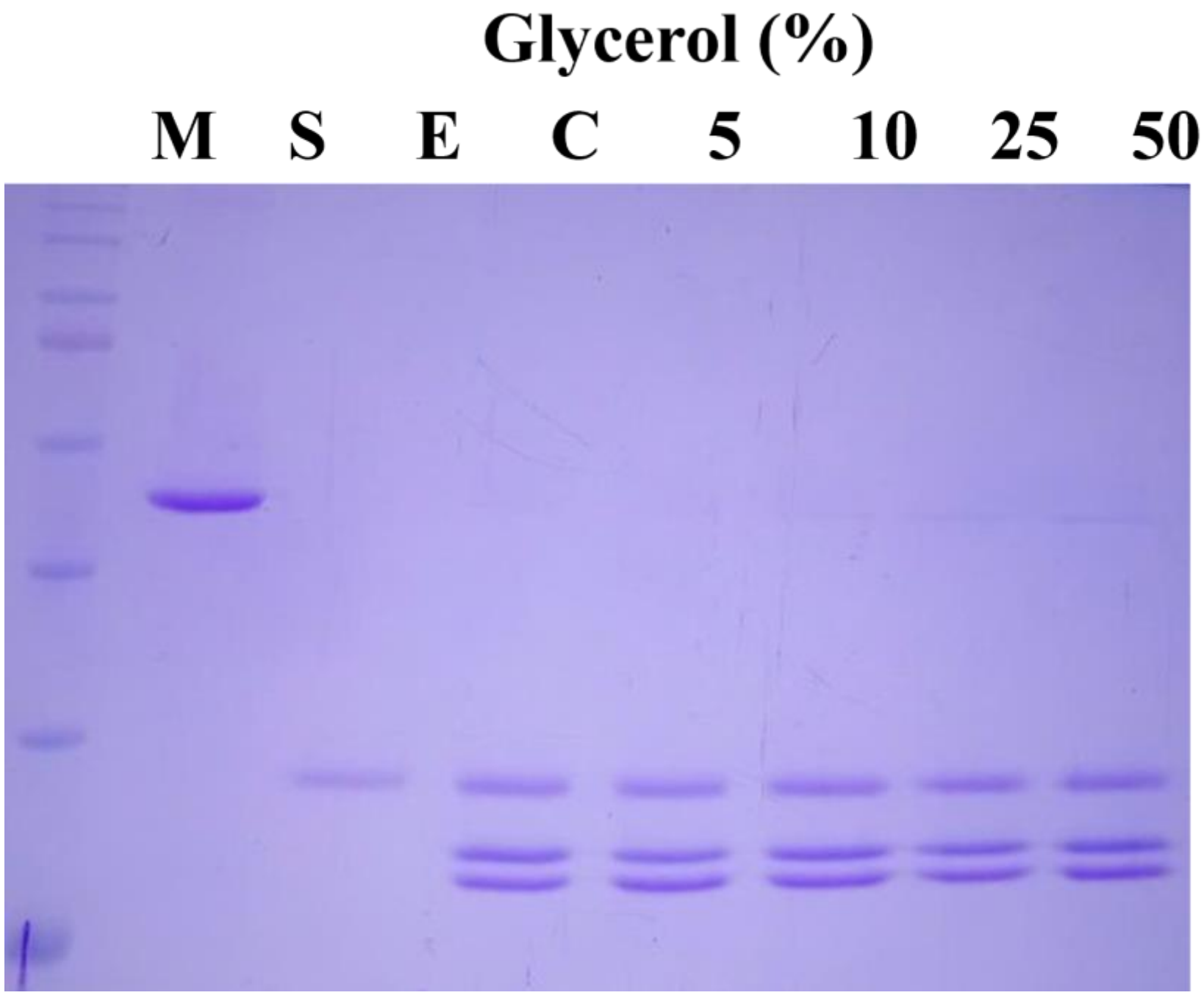

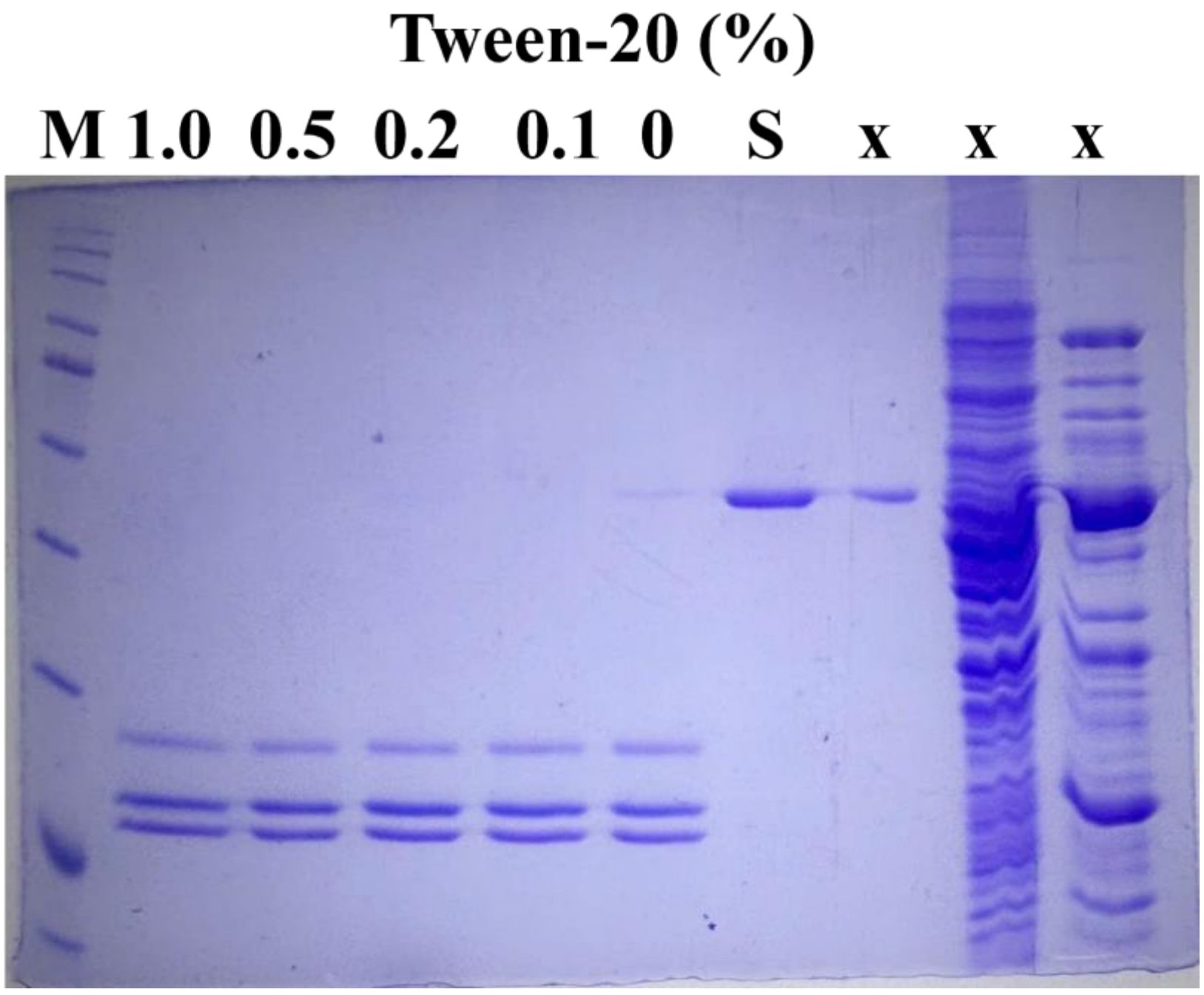

TritonX-100 (%)

M S 0 0.05 0.1 0.2 0.5

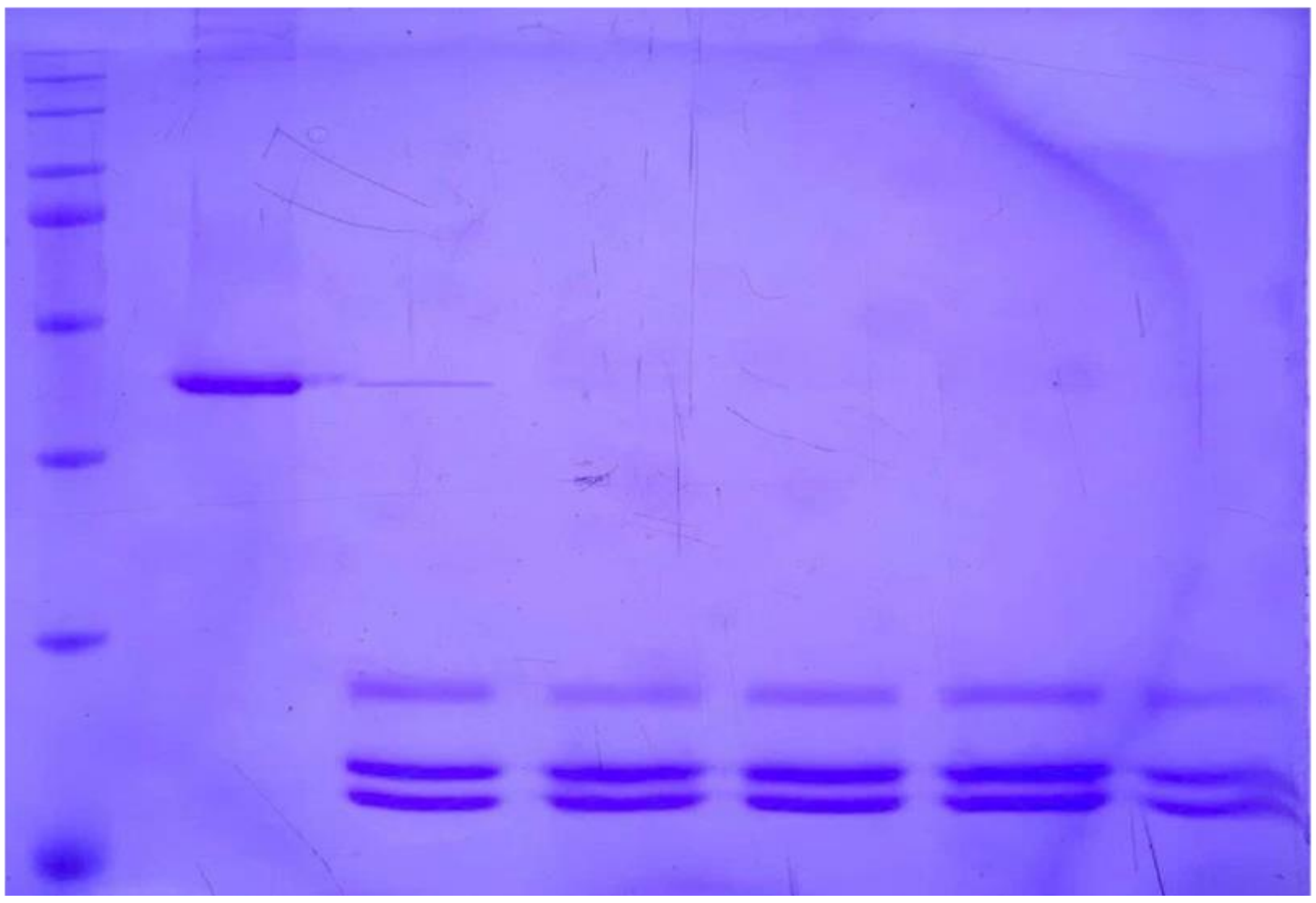

PMSF (mM)

M S 0 1 2 3 4 5

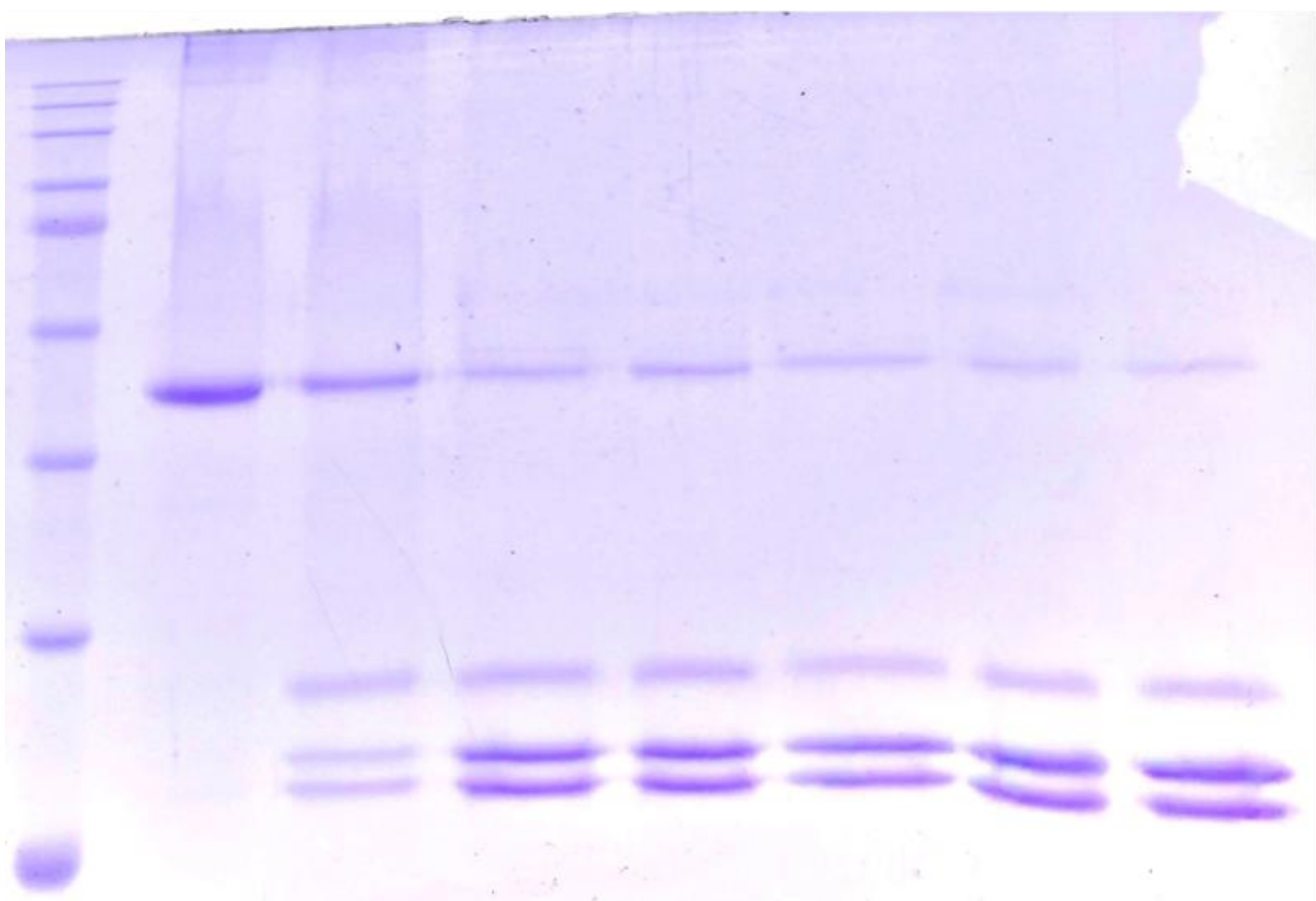

EDTA (mM)

x M S 0 1 2 3 4 5 x

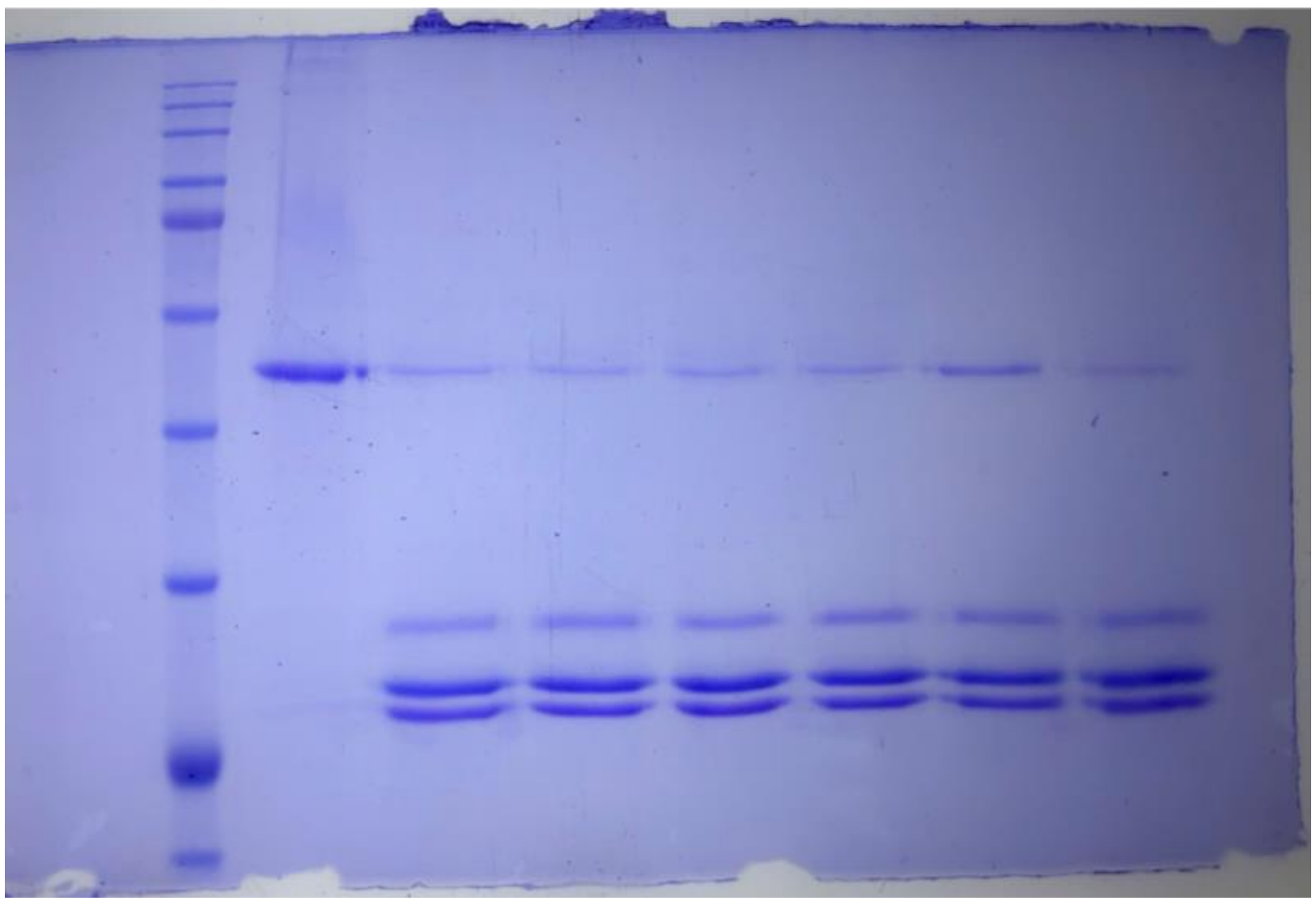

Denaturants

Urea (M)

x M S E 0 0.5 1.0 1.5 2.0 x

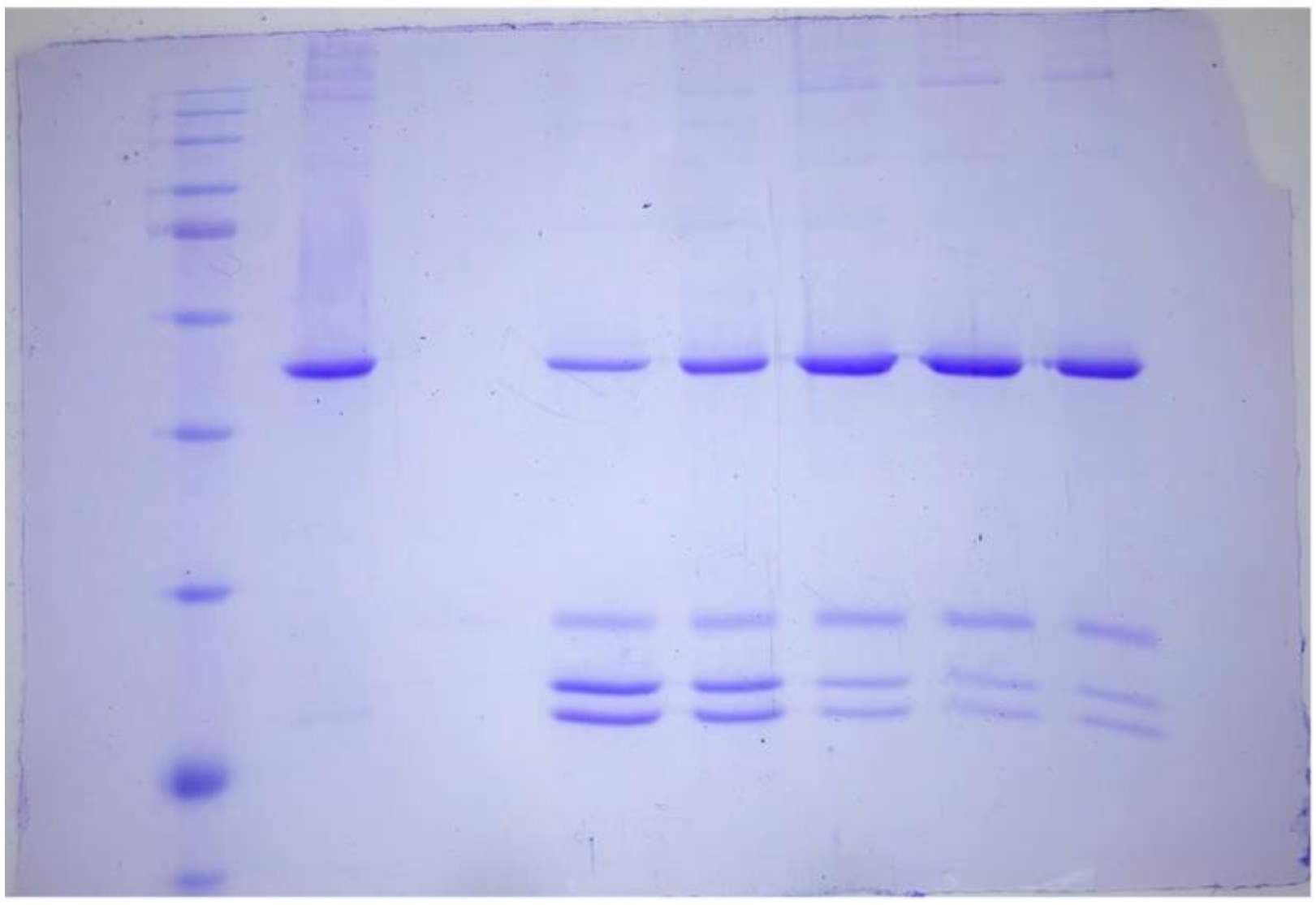

Guanidine-HCl (M)

x x M S 0 0.5 1.0 1.5 2.0 x

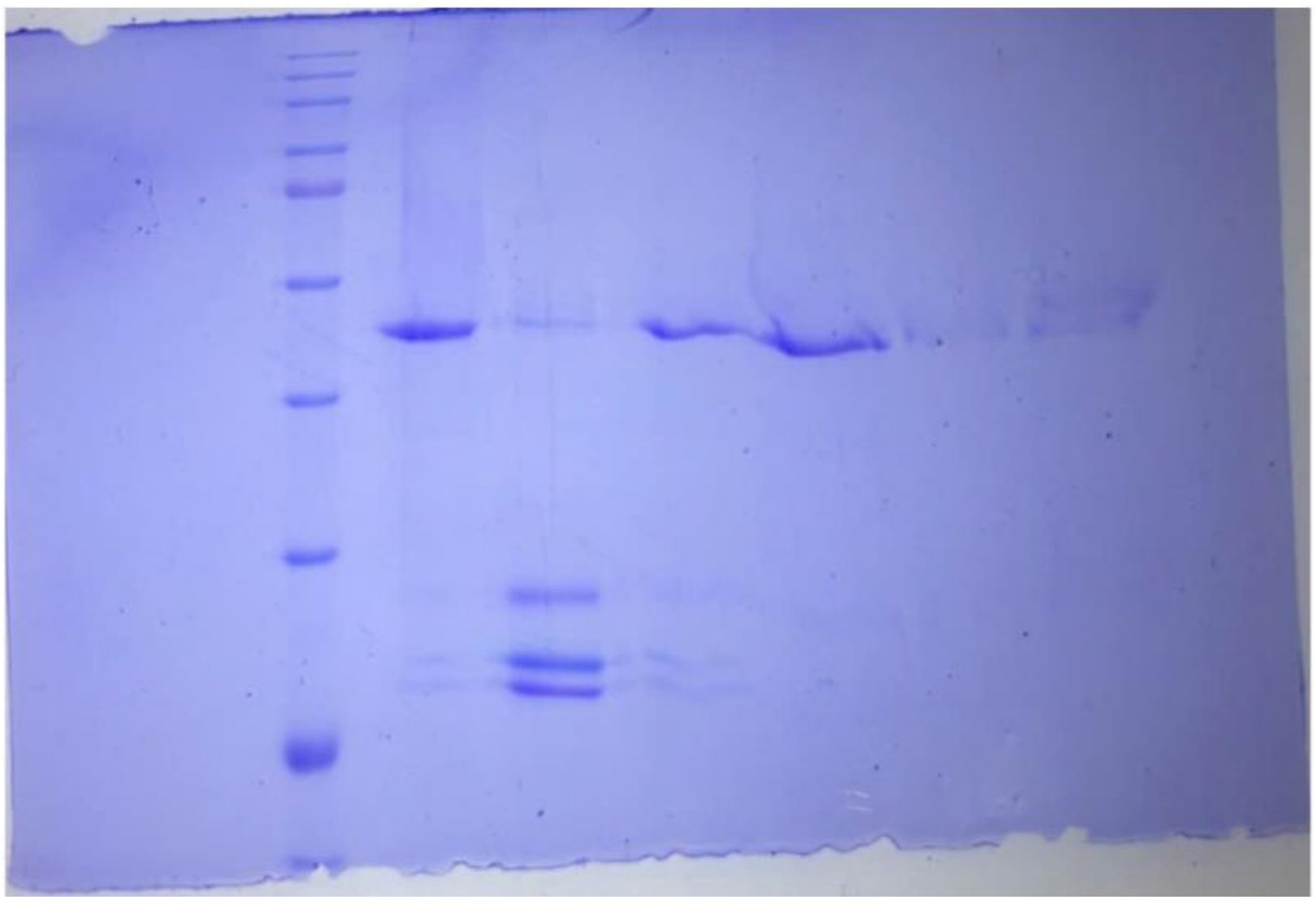

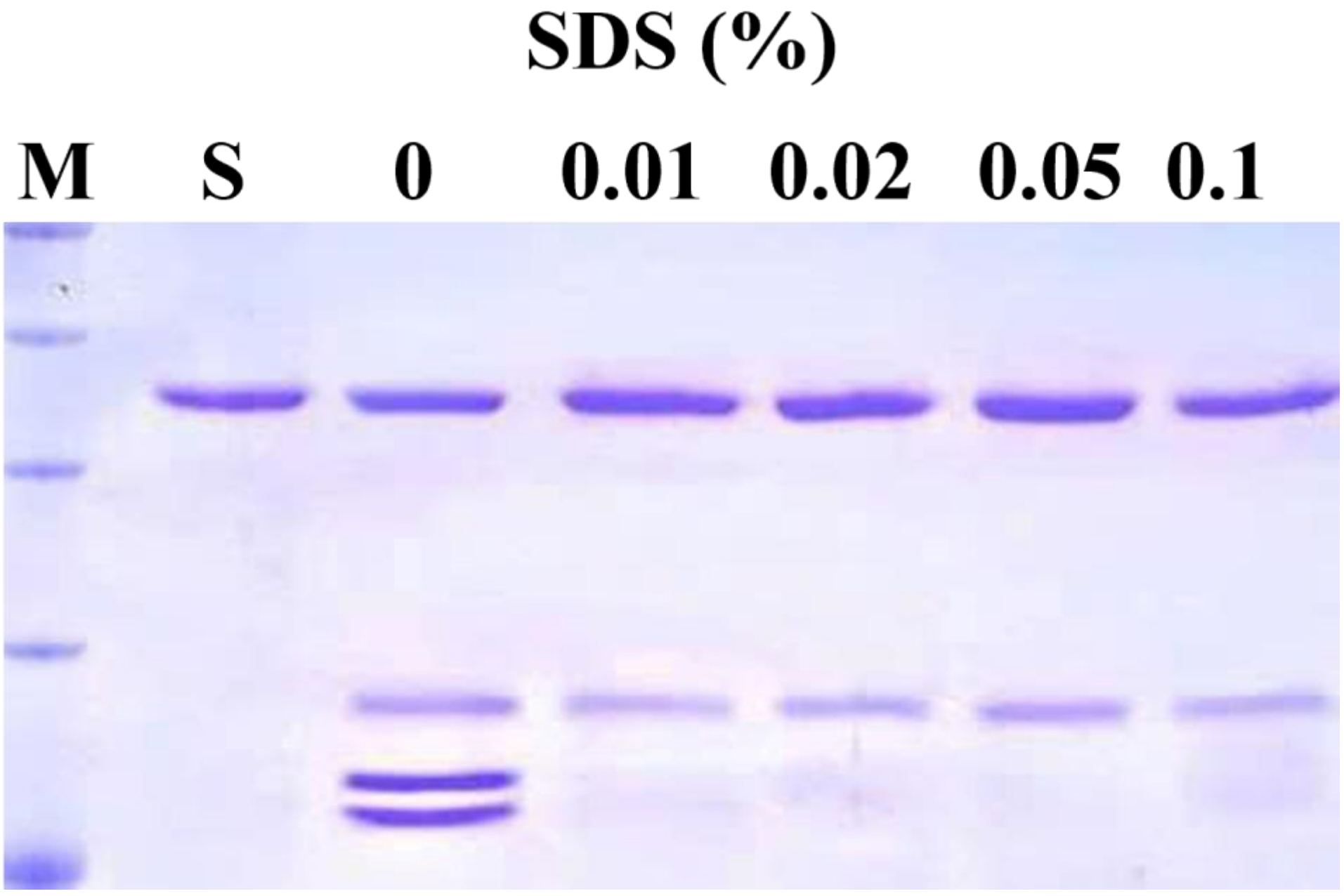
SDS (%)
M S 0 0.01 0.02 0.05 0.1

Reducing agents

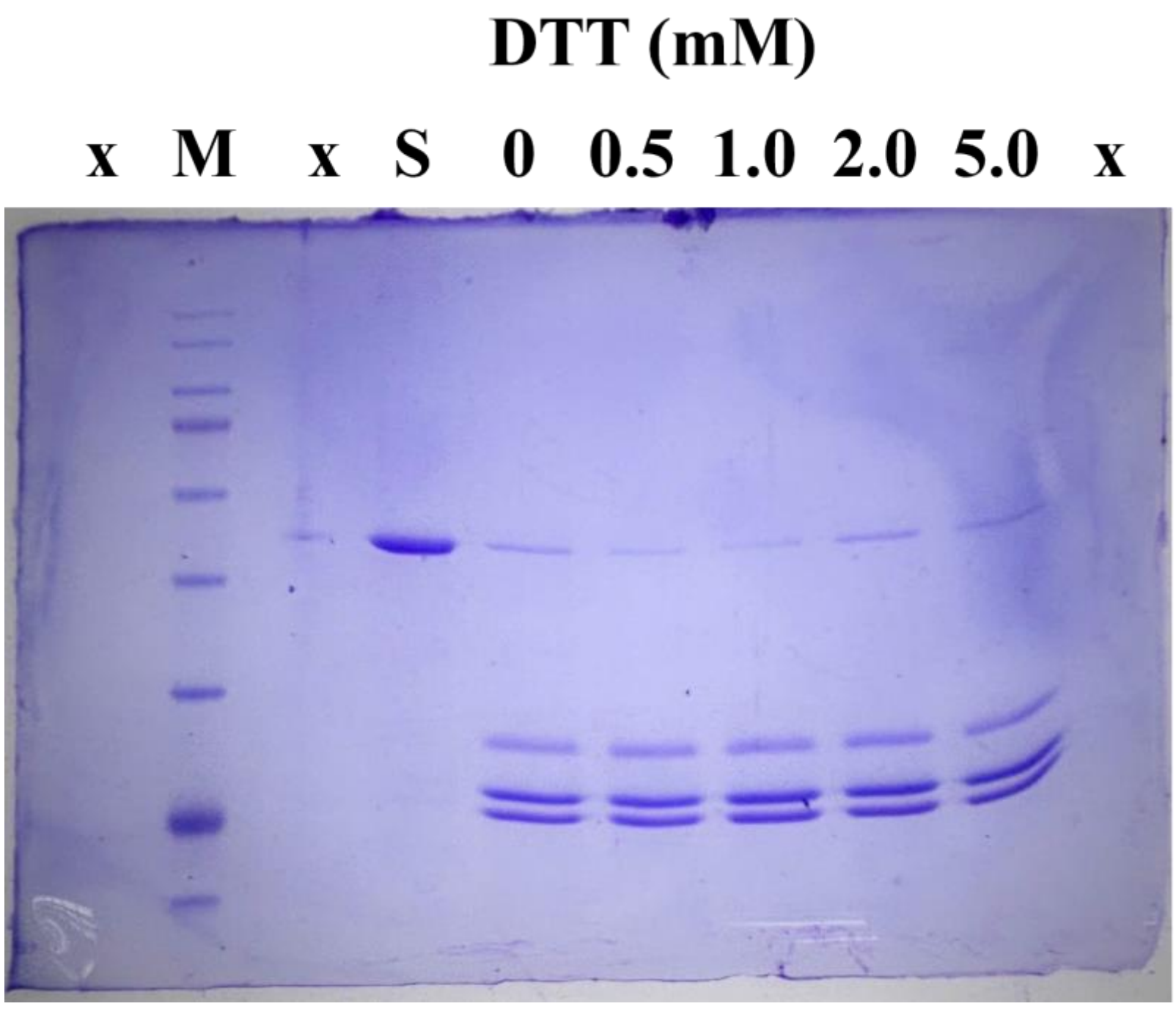

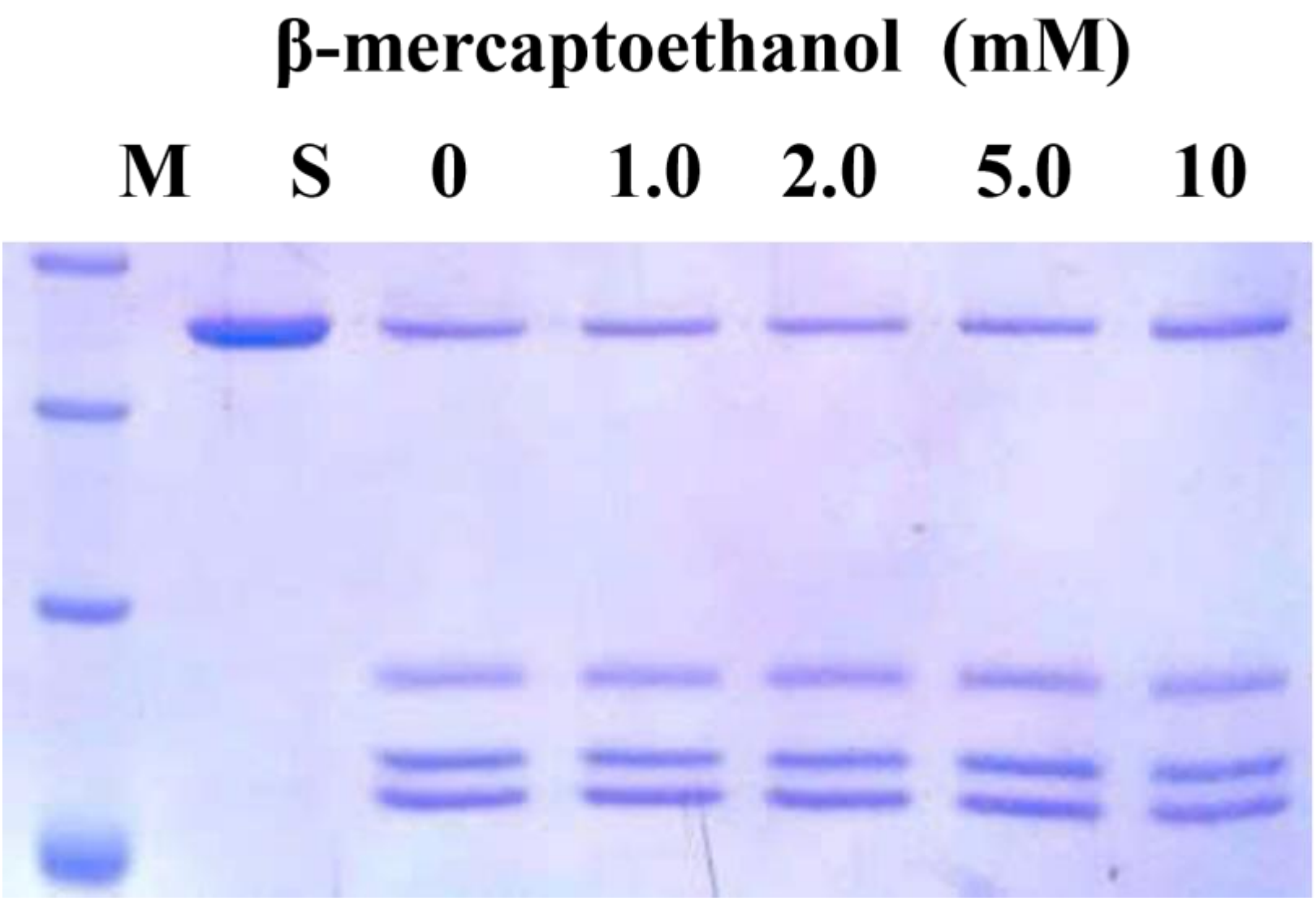

Fig 4. Effects of commonly used protein purification and stabilization reagents on 3CLpro cleavage activity

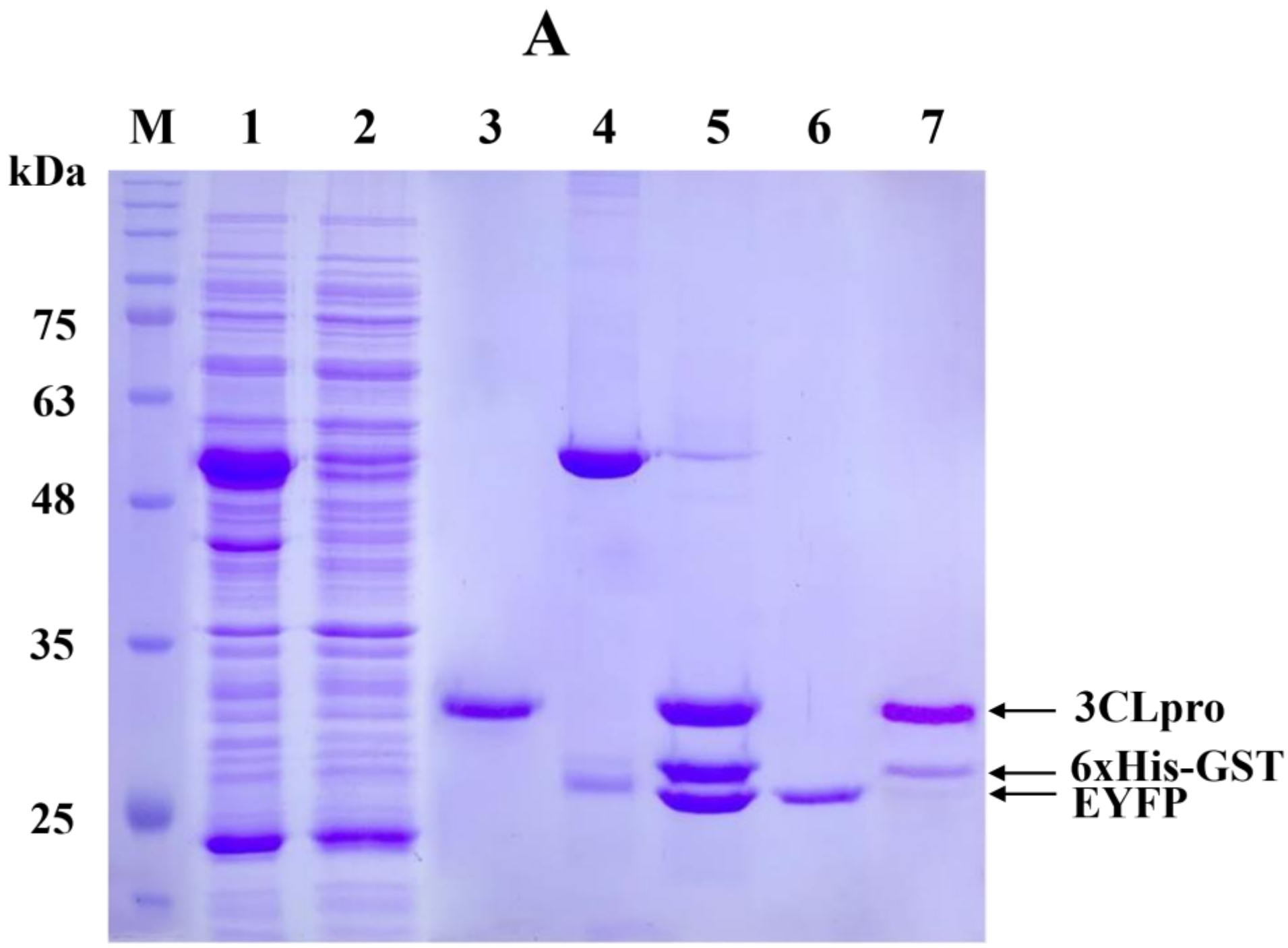
A
M
1
2
3
4
5
6
7
kDa
75
63
48
35
25
3CLpro
6xHis-GST
EYFP

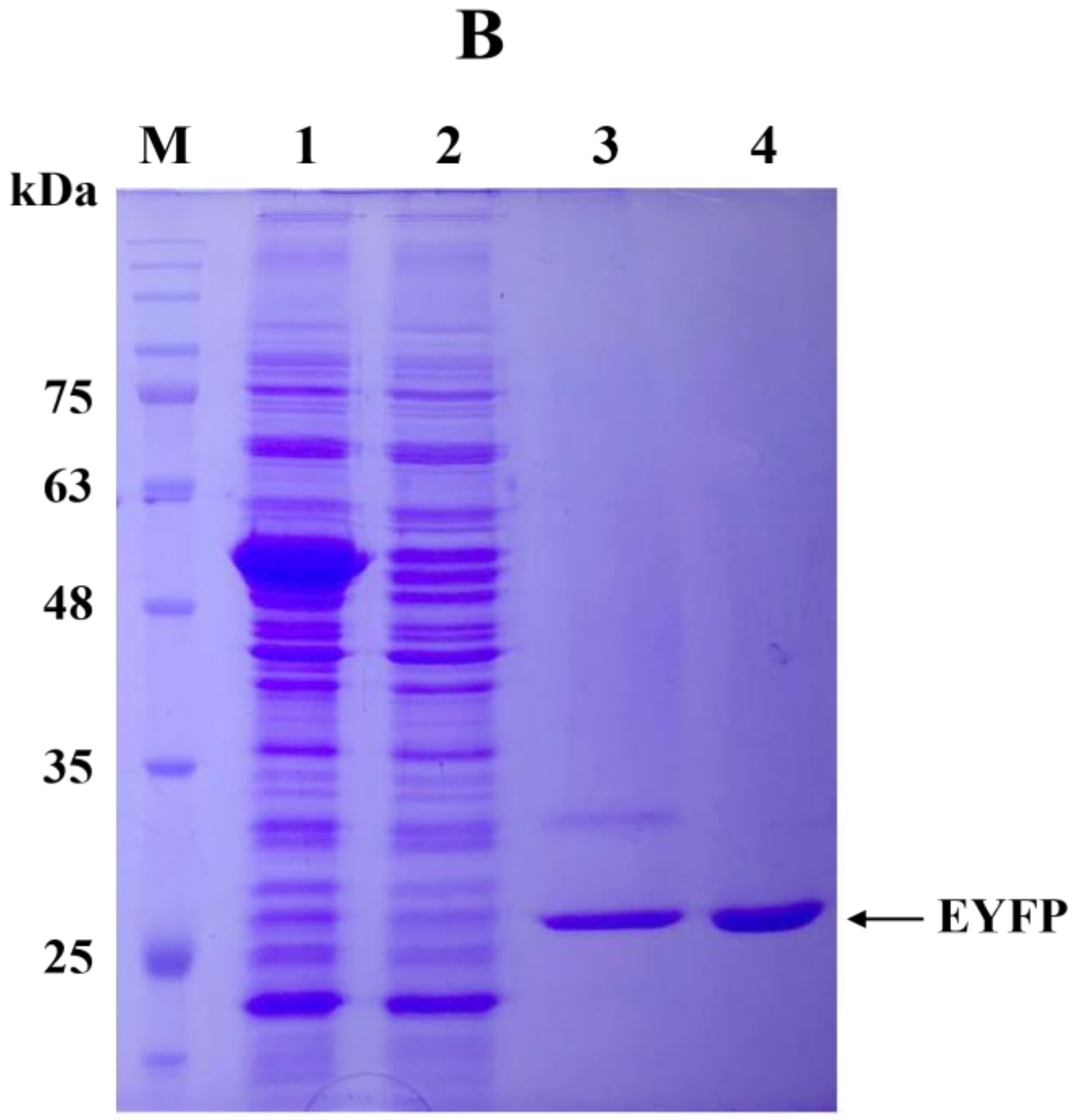
B
M
1
2
3
4
kDa
75
63
48
35
25
EYFP

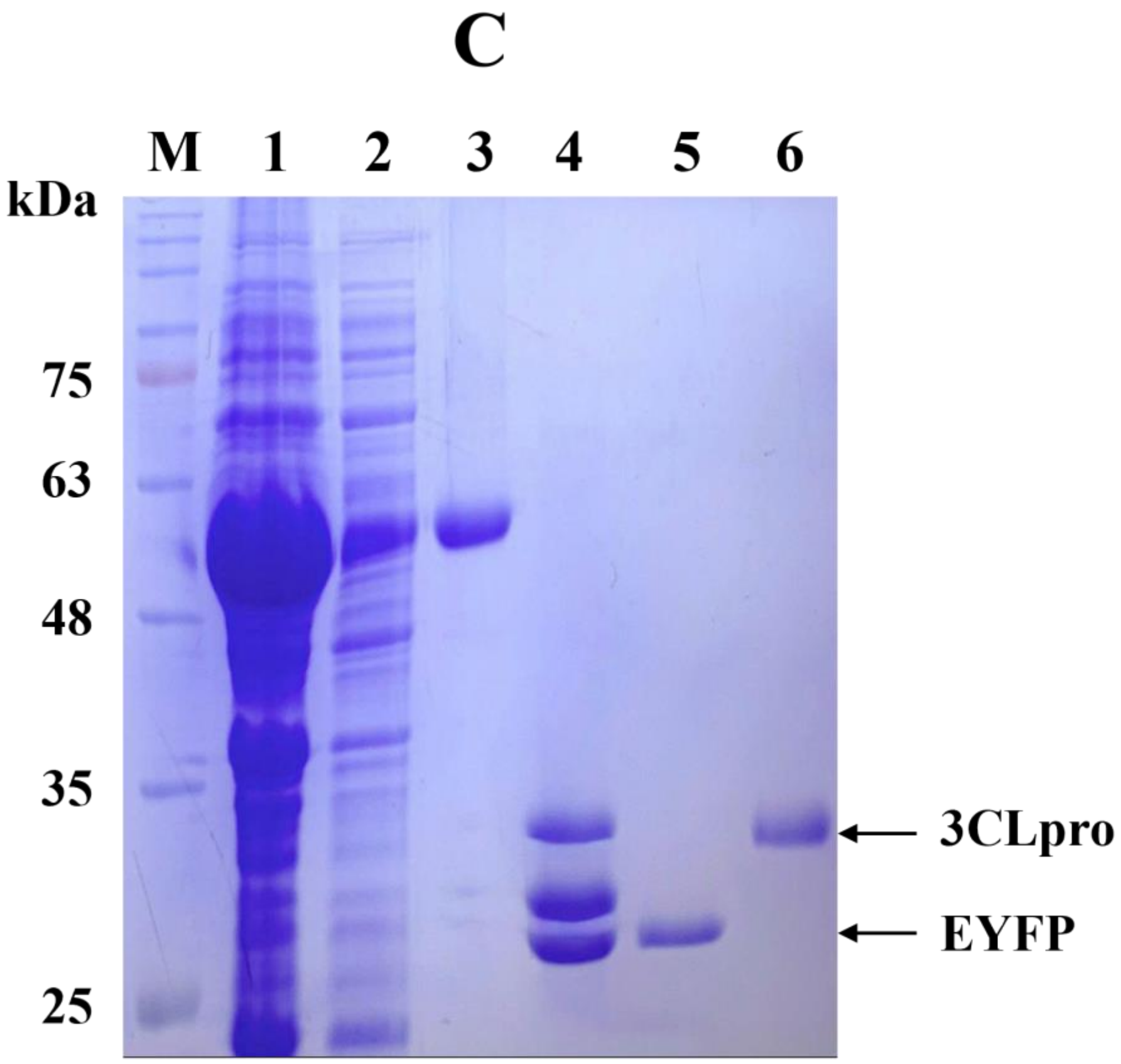

Fig 5. SDS–PAGE of 3CLPro cleaved products of 6×His–GST–AVLQSG–EYFP protein under in-solution (A), on-column (B) and during dialysis (C) cleavage conditions

B + C

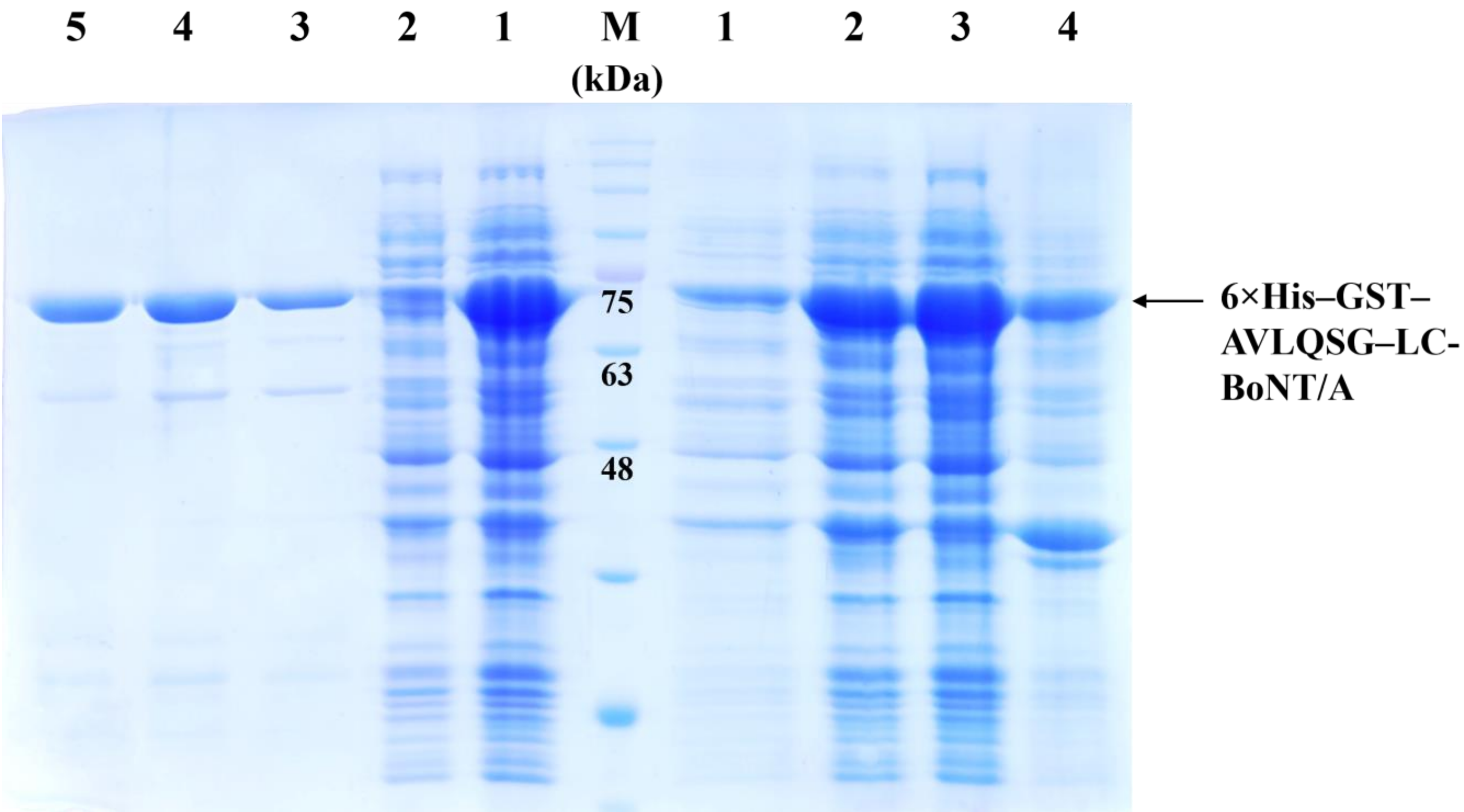

5 4 3 2 1 M 1 2 3 4
(kDa)
75
63
48
6×His–GST–AVLQSG–LC-BoNT/A

D

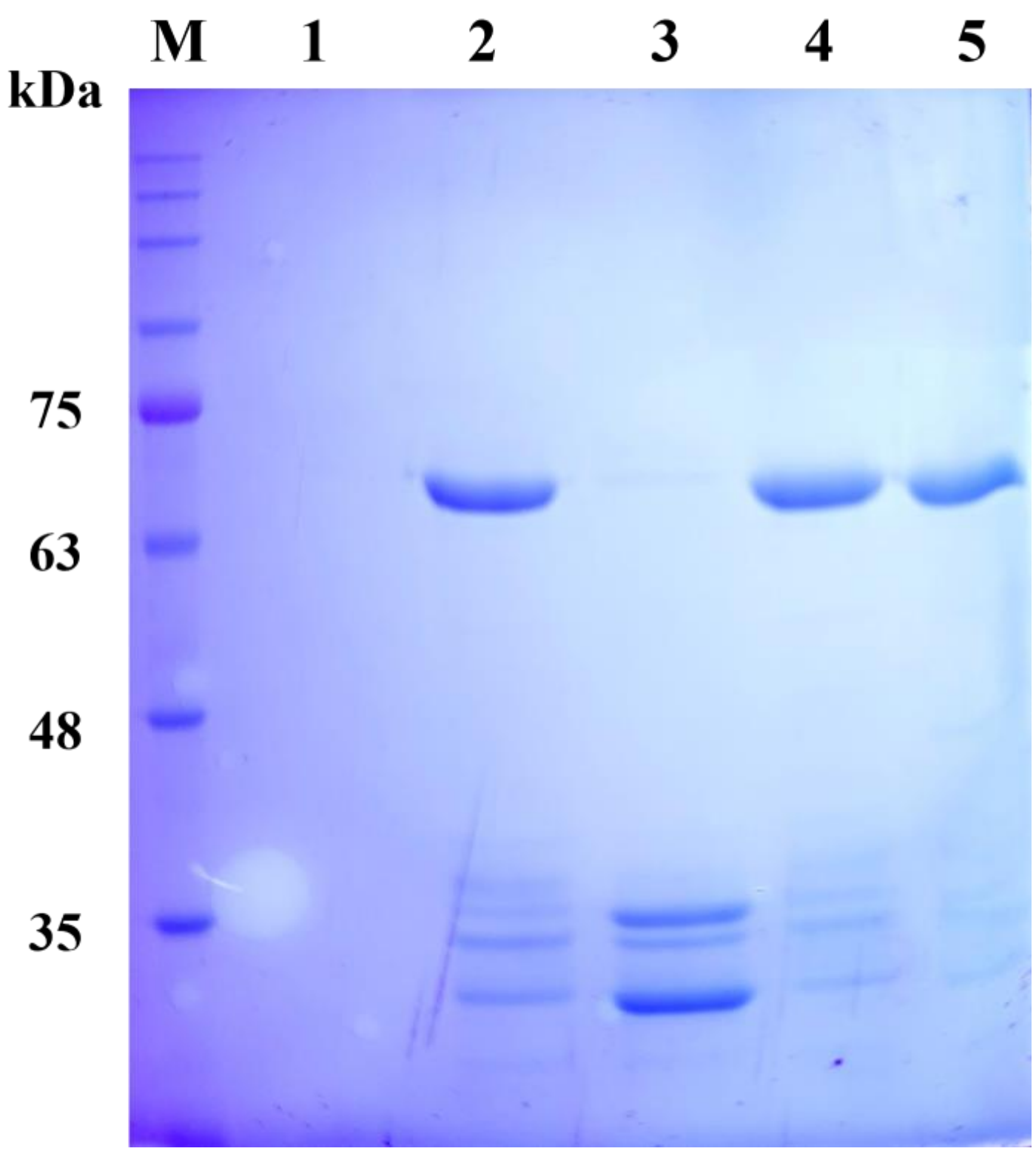

M 1 2 3 4 5
kDa
75
63
48
35

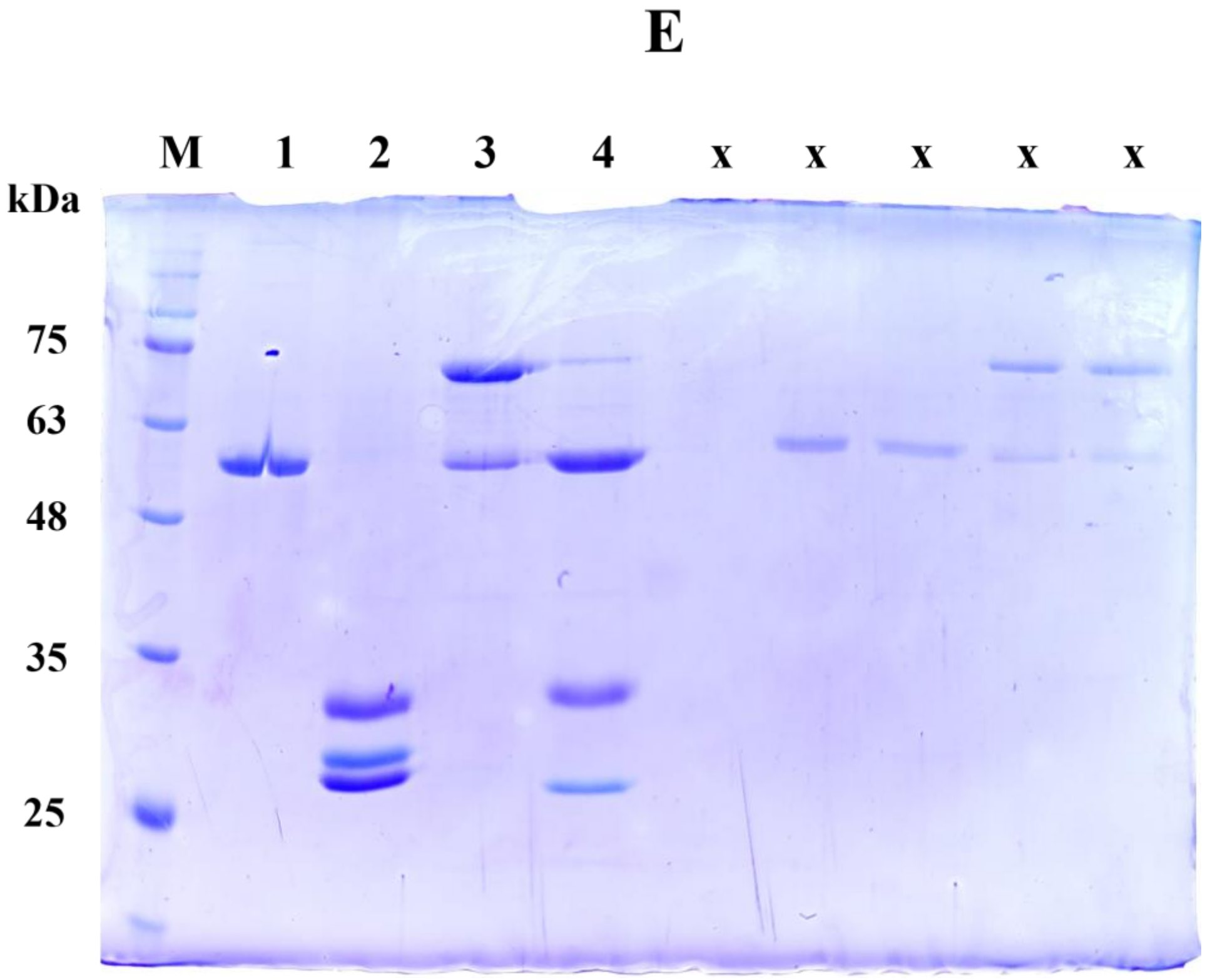

Fig 6. Schematic representation of the pET_RPLa vector and its application for expression of recombinant protein BoNT/A
