# Supplementary material for: SARS-CoV-2 3CLpro as an efficient endopeptidase for fusion tag removal in recombinant protein production
Source: PLoS One. 2026 Nov 11;21(11):e0362685. doi: 10.1371/journal.pone.0362685 (PMC13754225; doi:10.1371/journal.pone.0362685)
Supplement: Supplementary file 2 [file pone.0362685.s002.pdf]

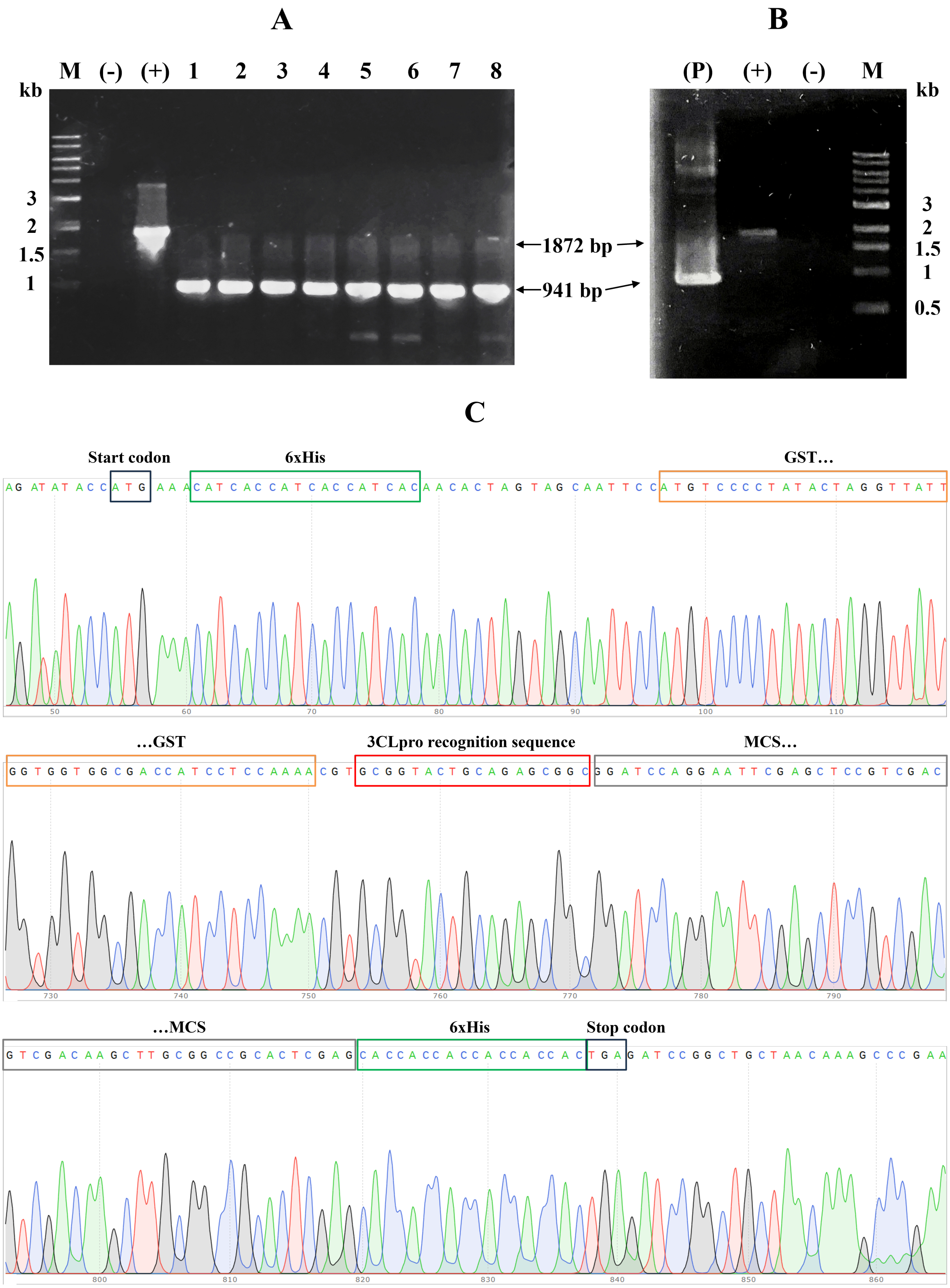
A
M (-) (+) 1 2 3 4 5 6 7 8
kb
3
2
1.5
1
1872 bp
941 bp
B
(P) (+) (-) M
kb
3
2
1.5
1
0.5
C
Start codon
6xHis
GST…
…GST
3CLpro recognition sequence
MCS…
…MCS
6xHis
Stop codon

D

Alignment with Reference Sequence – Database BLAST Nucleotide Alignment

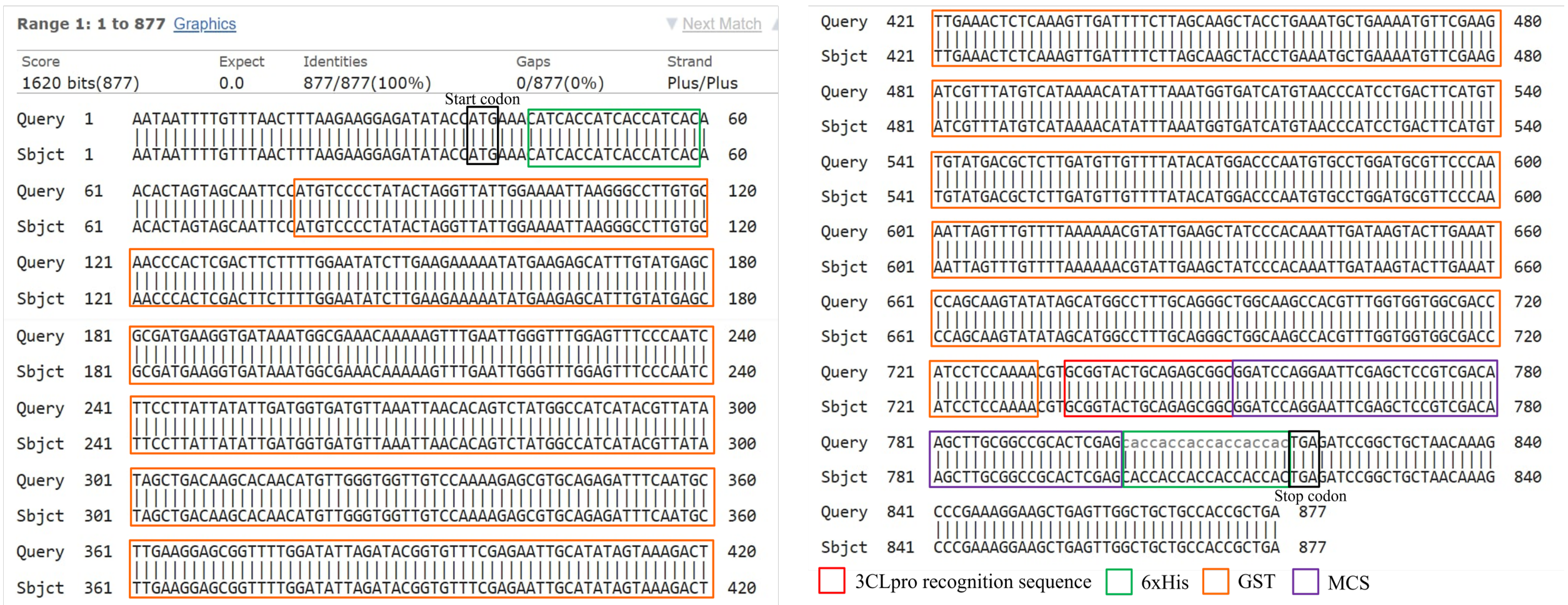

Fig S2. Verification of the pET_RPLa vector by PCR and Sanger sequencing

(A) Colony PCR products from pET_RPLa-transformed *E. coli* TOP10 colonies using the T7 promoter and T7 terminator primers. DNA ladder (lane M); negative control (lane (-)); positive control using pETM33-Nsp5-Mpro as the template (lane (+)); pET_RPLa-transformed colonies (lanes 1–8).

(B) PCR products from the purified pET_RPLa vector using the T7 promoter and T7 terminator primers. DNA ladder (lane M); negative control (lane (-)); positive control using pETM33-Nsp5-Mpro as the template (+)); PCR product amplified from purified pET_RPLa vector (lane P).

(C) Representative Sanger sequencing chromatogram of the pET_RPLa vector showing the 3CLpro recognition sequence and relevant vector elements.

(D) Alignment of the Sanger sequencing read with the designed pET_RPLa reference sequence.
