# Supplementary material for: SARS-CoV-2 3CLpro as an efficient endopeptidase for fusion tag removal in recombinant protein production
Source: PLoS One. 2026 Nov 11;21(11):e0362685. doi: 10.1371/journal.pone.0362685 (PMC13754225; doi:10.1371/journal.pone.0362685)
Supplement: Supplementary file 3 [file pone.0362685.s003.pdf]

ImageJ densitometry data underlying Figs 3 and 4

Band intensities were measured from Coomassie Brilliant Blue R-250-stained SDS-PAGE gels using ImageJ. Peak 1 represents the fusion substrate (~56 kDa), whereas Peaks 2 and 3 represent the cleavage products (~29 and ~27 kDa, respectively). Cleavage (%) was estimated as [(Peak 2 + Peak 3)/(Peak 1 + Peak 2 + Peak 3)] × 100. For Table S3.2, relative cleavage (%) was calculated as the ratio of the estimated cleavage percentage in the presence of each reagent to that of the corresponding control without the reagent. These values were used for semiquantitative comparisons.

Table S3.1. Densitometric analysis of cleavage under different reaction conditions (Fig. 3).

A. pH

Condition	Peak 1 area	Peak 2 area	Peak 3 area	Cleavage (%)
**pH 7.0**	910.113	2,507.811	4,930.225	89.10
**pH 7.3**	1,026.698	2,262.326	3,705.569	85.32
**pH 7.5**	4,246.376	1,253.548	2,359.740	45.97
**pH 7.8**	4,728.154	1,336.669	2,602.154	45.45
**pH 8.0**	4,514.619	1,348.083	2,413.912	45.45
**pH 8.3**	3,528.376	1,005.669	3,111.296	53.85
**pH 8.5**	4,106.326	179.941	1,092.326	23.65

B. Substrate-to-enzyme ratio and incubation time

Condition	Peak 1 area	Peak 2 area	Peak 3 area	Cleavage (%)
**S:E 3:1; 3 h**	9.536	2,111.790	3,436.598	99.83
**S:E 3:1; 6 h**	82.607	2,957.761	4,804.983	98.95
**S:E 3:1; 18 h**	117.607	2,567.740	3,960.841	98.23
**S:E 5:1; 3 h**	2,129.669	2,138.669	4,007.012	74.26
**S:E 5:1; 6 h**	216.263	1,689.477	3,541.648	96.03
**S:E 5:1; 18 h**	302.092	1,551.790	3,778.083	94.64
**S:E 10:1; 3 h**	4,170.205	1,262.083	2,741.598	48.98
**S:E 10:1; 6 h**	884.891	1,042.426	2,424.234	79.66
**S:E 10:1; 18 h**	595.648	1,395.033	3,719.912	89.57
**S:E 20:1; 3 h**	5,451.912	406.355	1,634.255	27.24
**S:E 20:1; 6 h**	2,199.770	244.163	1,517.305	44.47
**S:E 20:1; 18 h**	1,305.426	1,194.690	3,093.719	76.66

C. Temperature and incubation time

Condition	Peak 1 area	Peak 2 area	Peak 3 area	Cleavage (%)
**4°C; 3 h**	5,036.276	1,252.527	1,837.113	38.02
**4°C; 6 h**	1,829.134	1,115.113	1,809.527	61.52
**4°C; 18 h**	614.941	1,693.770	2,543.355	87.33
**25°C; 3 h**	644.941	3,032.749	3,487.163	91.00
**25°C; 6 h**	821.305	3,395.477	3,749.184	89.69
**25°C; 18 h**	699.648	2,925.891	3,379.113	90.01
**37°C; 3 h**	197.627	2,772.426	3,265.355	96.83
**37°C; 6 h**	125.385	2,051.426	3,003.598	97.58
**37°C; 18 h**	60.849	1,085.184	1,897.113	98.00

Table S3.2. Densitometric analysis of cleavage in the presence of different reagents (Fig. 4)

Imidazole

mM	Peak 1 area	Peak 2 area	Peak 3 area	Cleavage (%)	Relative cleavage (%)
**0**	3,508.790	4,061.075	3,442.154	68.14	100
**10**	4,133.690	4,804.024	3,687.619	67.26	98.71
**50**	7,373.418	2,157.962	1,868.012	35.32	51.83
**100**	8,920.125	942.234	764.648	16.06	23.57
**200**	6,563.983	390.042	306.971	9.60	14.09

Reduced glutathione

mM	Peak 1 area	Peak 2 area	Peak 3 area	Cleavage (%)	Relative cleavage (%)
**0**	4,265.134	6,039.347	5,712.033	73.37	100
**0.1**	3,479.134	5,515.134	5,667.447	76.27	103.95
**1.0**	3,551.497	6,965.447	6,967.861	79.69	108.61
**5.0**	10,894.406	1,241.477	1,591.477	20.64	28.13
**10**	10,745.347	360.627	466.991	7.15	9.75

Maltose

mM	Peak 1 area	Peak 2 area	Peak 3 area	Cleavage (%)	Relative cleavage (%)
**0**	3,097.326	12,535.004	12,090.439	88.83	100
**0.1**	2,882.326	14,186.196	12,686.681	90.31	101.67
**1.0**	2,592.397	10,521.832	9,586.610	88.58	99.72
**5.0**	2,368.326	15,637.246	14,236.731	92.65	104.31
**10**	1,783.569	15,707.777	3,936.539	91.68	103.21

NaCl

mM	Peak 1 area	Peak 2 area	Peak 3 area	Cleavage (%)	Relative cleavage (%)
**0**	2,494.030	10,548.210	9,672.330	89.02	100
**150**	721.010	10,855.550	10,916.860	96.79	108.73
**300**	1,118.620	12,693.790	13,211.650	95.86	107.68
**600**	836.130	12,193.140	12,024.520	96.66	108.59
**800**	1,341.550	12,044.520	10,360.170	94.35	105.99

KCl

**mM**	**Peak 1 area**	**Peak 2 area**	**Peak 3 area**	**Cleavage (%)**	**Relative cleavage (%)**
**0**	3,135.539	13,456.037	17,756.794	90.87	100
**150**	3,005.711	12,862.894	16,414.723	90.69	99.80
**300**	2,541.225	14,919.208	17,655.593	92.76	102.08
**400**	1,955.447	15,034.765	16,600.915	94.18	103.64
**600**	754.062	12,464.522	16,838.832	97.49	107.28

$CaCl_2$

**mM**	**Peak 1 area**	**Peak 2 area**	**Peak 3 area**	**Cleavage (%)**	**Relative cleavage (%)**
**0**	12,031.368	3,320.347	3,568.740	36.41	100
**1.0**	13,662.439	4,340.782	4,521.912	39.35	108.06
**2.0**	11,567.782	5,477.803	6,669.267	51.22	140.68
**5.0**	9,563.489	6,186.510	6,879.439	57.74	158.58
**10**	3,528.882	5,712.903	7,130.690	78.45	215.45

$MgCl_2$

**mM**	**Peak 1 area**	**Peak 2 area**	**Peak 3 area**	**Cleavage (%)**	**Relative cleavage (%)**
**0**	11,346.150	1,663.912	2,755.790	28.03	100
**1.0**	11,789.340	2,749.640	4,217.397	37.14	132.50
**5.0**	10,075.170	2,571.276	4,316.660	40.61	144.85
**10**	6,542.711	3,077.054	6,450.874	59.29	211.49
**25**	4,368.489	1,806.983	4,869.690	60.45	215.63

$MgSO_4$

**mM**	**Peak 1 area**	**Peak 2 area**	**Peak 3 area**	**Cleavage (%)**	**Relative cleavage (%)**
**0**	9,855.146	1,976.326	2,858.882	32.91	100
**1.0**	8,295.095	2,691.861	3,901.610	44.29	134.55
**5.0**	9,404.874	2,912.276	4,141.832	42.86	130.21
**10**	8,153.903	2,390.004	3,653.953	42.57	129.33
**25**	4,836.761	2,437.569	2,798.134	51.98	157.93

$ZnSO_4$

mM	Peak 1 area	Peak 2 area	Peak 3 area	Cleavage (%)	Relative cleavage (%)
**0**	12,425.581	1,973.933	2,688.276	27.28	100
**0.1**	11,414.874	211.092	815.234	8.25	30.24
**0.5**	10,577.317	112.485	697.163	7.11	26.06
**1.0**	9,064.439	151.607	313.971	4.89	17.91
**10**	6,233.539	58.192	263.142	4.90	17.97

Glycerol

%	Peak 1 area	Peak 2 area	Peak 3 area	Cleavage (%)	Relative cleavage (%)
**0**	134.231	6,065.740	5,302.719	98.83	100
**5**	6.121	5,047.255	6,876.426	99.95	101.13
**10**	236.385	5,687.962	5,761.548	97.98	99.13
**25**	341.213	4,584.598	4,783.548	96.49	97.62
**50**	487.678	5,376.962	5,518.497	95.72	96.85

Tween-20

%	Peak 1 area	Peak 2 area	Peak 3 area	Cleavage (%)	Relative cleavage (%)
**0**	1,299.184	10,725.832	9,954.518	94.09	100
**0.1**	0.000	13,310.439	11,889.832	100.00	106.28
**0.2**	0.000	14,367.388	12,487.196	100.00	106.28
**0.5**	0.000	12,211.803	11,265.660	100.00	106.28
**1.0**	0.000	12,905.329	12,339.359	100.00	106.28

Triton X-100

%	Peak 1 area	Peak 2 area	Peak 3 area	Cleavage (%)	Relative cleavage (%)
**0**	187.263	5,446.640	4,852.912	98.21	100
**0.05**	0.000	5,408.175	5,287.497	100.00	101.82
**0.1**	0.000	5,898.368	4,888.326	100.00	101.82
**0.2**	0.000	6,009.125	5,149.740	100.00	101.82
**0.5**	0.000	2,926.468	2,396.669	100.00	101.82

PMSF

mM	Peak 1 area	Peak 2 area	Peak 3 area	Cleavage (%)	Relative cleavage (%)
**0**	8,405.903	3,729.468	4,153.660	48.40	100
**1.0**	3,672.296	10,863.823	10,637.681	85.41	176.49
**2.0**	3,735.882	11,528.146	11,159.267	85.86	177.42
**3.0**	2,592.104	12,854.690	10,876.761	90.15	186.28
**4.0**	2,023.347	11,955.380	9,035.581	91.21	188.47
**5.0**	1,898.154	16,253.317	12,945.832	93.90	194.02

EDTA

mM	Peak 1 area	Peak 2 area	Peak 3 area	Cleavage (%)	Relative cleavage (%)
**0**	1,607.033	6,822.581	5,726.468	88.65	100
**1.0**	1,072.669	7,244.681	6,080.882	92.55	104.40
**2.0**	1,058.305	7,435.702	5,907.054	92.65	104.52
**3.0**	1,115.548	7,872.560	6,462.983	92.78	104.66
**4.0**	1,884.205	8,190.045	7,089.054	89.02	100.42
**5.0**	549.406	10,199.530	8,677.196	97.17	109.62

β-mercaptoethanol

mM	Peak 1 area	Peak 2 area	Peak 3 area	Cleavage (%)	Relative cleavage (%)
**0**	6,180.711	7,098.539	8,086.489	71.07	100
**1.0**	5,880.397	7,460.953	7,316.175	71.53	100.65
**2.0**	5,108.104	6,572.246	7,451.953	73.30	103.14
**5.0**	5,880.205	7,504.489	7,996.882	72.50	102.01
**10**	6,557.075	5,279.731	4,992.740	61.04	85.88

DTT

mM	Peak 1 area	Peak 2 area	Peak 3 area	Cleavage (%)	Relative cleavage (%)
**0**	1,651.426	9,720.246	9,362.539	92.04	100
**0.5**	698.770	10,391.660	9,969.125	96.68	105.05
**1.0**	668.698	12,164.045	11,117.125	97.21	105.62
**2.0**	2,016.497	12,206.459	10,992.953	92.00	99.97
**5.0**	1,632.640	9,858.543	3,021.933	88.75	96.43

Urea

Condition	Peak 1 area	Peak 2 area	Peak 3 area	Cleavage (%)	Relative cleavage (%)
**0**	4,013.912	4,940.820	4,678.912	70.56	100
**0.5**	5,595.447	3,479.113	3,529.062	55.60	78.81
**1.0**	8,797.175	1,655.284	1,734.113	27.81	39.42
**1.5**	10,069.004	957.891	839.042	15.14	21.46
**2.0**	10,836.175	786.598	750.062	12.42	17.60

Note: At higher concentrations of $CaCl_2$ (10 mM), $MgCl_2$ (10-25 mM), and $MgSO_4$ (25 mM), both substrate and cleavage-product bands showed reduced intensities despite high calculated relative cleavage values. These changes may reflect protein precipitation or loss, potentially leading to an overestimation of relative cleavage. Guanidine hydrochloride caused marked changes in protein band intensities and patterns on SDS-PAGE, preventing reliable densitometric analysis. In the presence of SDS (0.01-0.1%), cleavage-product bands were not detectable, precluding reliable quantification using ImageJ.
